# Deterministic and Stochastic Models of *Arabidopsis thaliana* Flowering

**DOI:** 10.1007/s11538-018-0528-x

**Published:** 2018-11-08

**Authors:** E. Haspolat, B. Huard, M. Angelova

**Affiliations:** 1 Department of Mathematics and Information Sciences, Northumbria University, Newcastle upon Tyne, NE1 8ST UK; 2 School of Information Technology, Deakin University, Melbourne Burwood Campus, Burwood, VIC 3125 Australia

**Keywords:** Arabidopsis flowering, Gene regulatory network, Deterministic–stochastic linear stability, Ordinary delay differential equations

## Abstract

Experimental studies of the flowering of *Arabidopsis thaliana* have shown that a large complex gene regulatory network (GRN) is responsible for its regulation. This process has been mathematically modelled with deterministic differential equations by considering the interactions between gene activators and inhibitors (Valentim et al. in PLoS ONE 10(2):e0116973, 2015; van Mourik et al. in BMC Syst Biol 4(1):1, 2010). However, due to complexity of the model, the properties of the network and the roles of the individual genes cannot be deducted from the numerical solution the published work offers. Here, we propose simplifications of the model, based on decoupling of the original GRN to motifs, described with three and two differential equations. A stable solution of the original model is sought by linearisation of the original model which contributes to further investigation of the role of the individual genes to the flowering. Furthermore, we study the role of noise by introducing and investigating two types of stochastic elements into the model. The deterministic and stochastic nonlinear dynamic models of Arabidopsis flowering time are considered by following the deterministic delayed model introduced in Valentim et al. (2015). Steady-state regimes and stability of the deterministic original model are investigated analytically and numerically. By decoupling some concentrations, the system was reduced to emphasise the role played by the transcription factor *Suppressor of Overexpression of Constants1* ($\textit{SOC}1$) and the important floral meristem identity genes, *Leafy* ($\textit{LFY}$) and *Apetala1* ($\textit{AP}1$). Two-dimensional motifs, based on the dynamics of $\textit{LFY}$ and $\textit{AP}1$, are obtained from the reduced network and parameter ranges ensuring flowering are determined. Their stability analysis shows that $\textit{LFY}$ and $\textit{AP}1$ are regulating each other for flowering, matching experimental findings. New sufficient conditions of mean square stability in the stochastic model are obtained using a stochastic Lyapunov approach. Our numerical simulations demonstrate that the reduced models of Arabidopsis flowering time, describing specific motifs of the GRN, can capture the essential behaviour of the full system and also introduce the conditions of flowering initiation. Additionally, they show that stochastic effects can change the behaviour of the stability region through a stability switch. This study thus contributes to a better understanding of the role of $\textit{LFY}$ and $\textit{AP}1$ in Arabidopsis flowering.

## Introduction

*Arabidopsis thaliana* is a small, annual flowering plant in the Brassicaceae (mustard) family which is a favourite model organism for plant biology research due mainly to its small size, simple genome and rapid life cycle. The transition from vegetative to reproductive development, which is an initiation of flower growth, is crucial for the life cycle of any angiosperm plant like *Arabidopsis thaliana* (Krizek and Fletcher 2005; Ó’Maoiléidigh et al. 2014; Wang et al. 2014) as flowering on time is a key factor to achieve reproductivity of these plants. Physiological and environmental conditions of the plant regulate the timing of transition for the optimal reproductive achievement, and their reactions are integrated into a complex GRN which monitors and regulates this transition (Kardailsky et al. 1999; Levy and Dean 1998; Wellmer and Riechmann 2010). Genes and their regulatory interactions are significant factors in biological systems at the molecular level since the understanding of their impact on each other’s regulation is crucial to comprehend the response of gene disturbances on flowering time (Valentim et al. 2015). Recently, the dynamics of Arabidopsis flowering time regulation has been studied using a systems approach along with experimental data to understand the effect of the genes on flowering of *Arabidopsis thaliana* (Daly et al. 2009; Jaeger et al. 2013; Pullen et al. 2013; Valentim et al. 2015; Wang et al. 2014).

Numerous genes appear to be acting as flowering time regulators of *Arabidopsis thaliana* (Ryan et al. 2015), and different pathways have been constructed to reveal the flowering of this plant (Amasino 2010; Greenup et al. 2009; Kardailsky et al. 1999; Yant et al. 2009). This complex network of many interacting genes can be dynamically modelled using systems with many equations (Jaeger et al. 2013; Valentim et al. 2015; van Mourik et al. 2010; Wang et al. 2014). In this study, we consider the deterministic dynamic model of delay differential equations (DDEs) describing the flowering of the Arabidopsis species proposed by Valentim et al. (2015). This model involves core set of gene–regulator interactions, while protein–protein interactions are not explicitly included. The model is based on a feedback loop, constructed with eight genes, where six of them are internal: *Apetala1* (*AP1*), *Leafy* (*LFY*), *Suppressor of Overexpression of Constants 1* (*SOC1*), *Agamous-Like 24* (*AGL24*), *Flowering Locus T* (*FT*) and *FD*. The other two genes are considered as external inputs: *Short Vegetative Phase* (*SVP*) and *Flowering Locus C* (*FLC*).

System behaviour of the GRNs usually cannot be understood heuristically due to the complexity of interactions in organisms. We propose a different approach by simplifying the network and studying its behaviour. Stability analysis is used to study the properties of the GRN and threshold in flowering. Moreover, such analysis provides a reliability test and more insights into the behaviour of GRN’s elements.

Our stability analysis produces conditions which include the biological parameters. Such parameters are difficult to determine from the experiment, and one of our aims was to provide specific ranges for individual coefficients that secure stable solutions. To overcome this issue of complexity, we reduce the differential equation system by decoupling some concentrations before simplifying the new system using network motifs that capture essential characteristics of the floral transition. Examples of reduced *Arabidopsis thaliana* GRNs can be seen in the study of Pullen et al. (2013), where a complex flowering time pathway included in the model of Jaeger et al. (2013) was simplified by focusing on essential flowering genes. Following these papers, we produce a subsystem of our network with three different motifs.

Indeed, it is known that the floral meristem identity genes have an important role to control the floral meristem specification while the flower development process is starting (Irish 2010; Levy and Dean 1998; Simon et al. 1996). Thus, this minimal regulatory network consists of the main floral meristem identity genes of *Arabidopsis thaliana*: $\textit{AP}1$, $\textit{LFY}$, $\textit{FT}$ and $\textit{FD}$ where $\textit{AP}1$ is the dominant regulatory concentration of floral initiation with $\textit{LFY}$ in *Arabidopsis thaliana* (Irish 2010; Wellmer and Riechmann 2010) and has a key role between floral induction to flower formation, being a junction of flowering in the GRN (Kaufmann et al. 2010). On the other hand, $\textit{FT}$ induces flowering of Arabidopsis as an inhibitor and acts similarly with $\textit{LFY}$. Additionally, activation tagging isolates it (Kardailsky et al. 1999). Moreover, $\textit{FT}$ and transcription factor $\textit{FD}$ affect each other in the meristem as a combined activator (Wang et al. 2014). The aim of this subsystem is to construct parameter-dependent stability conditions that reflect essential behaviour of the complex network.

Another aim of this study is to investigate the properties of the simplified Arabidopsis flowering model modified with stochastic perturbations. The motifs are reflecting the essential behaviour of the complex network and can capture the significant behaviour of the full Arabidopsis flowering model and can investigate necessary conditions (threshold values of the concentrations) for the flowering initiation. The advantage of this approach is based on the realistic description of gene effects and their interactions on flowering of Arabidopsis. New sufficient conditions of mean square stability are obtained analytically for this simplified model using Lyapunov function. Analytical and numerical investigations of the stability are performed with respect to concentrations and noise terms.

This paper is organised as follows: in Sect. 2, the main features of the deterministic dynamic model of Arabidopsis flowering introduced in Valentim et al. (2015) are recalled, and analytical and numerical investigations of its steady state are both conducted. Section 3 provides a simplified deterministic model by decoupling some concentrations in the full model. A comparative numerical investigation of both models is also given. Deterministic motifs of the simplified model are presented in Sect. 4 along with an analytical investigation of their steady state and their stability. Stochastic perturbations of the motifs are investigated in Sect. 5 using Lyapunov functions to obtain sufficient conditions for their mean square stability. Finally, our concluding remarks are given in Sect. 6, while further technical information can be seen in “Appendix”.

## Deterministic Model

**Fig. 1:**
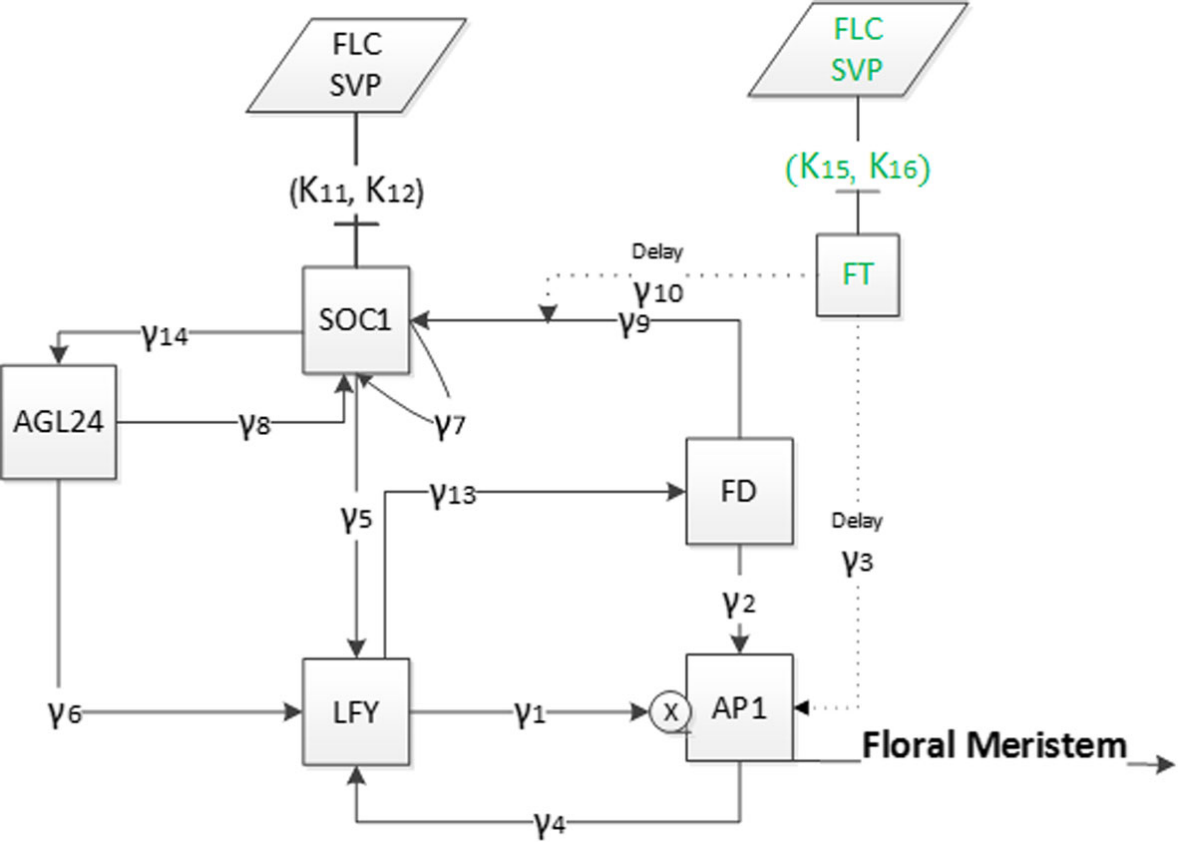
Flowchart of the model. Green and black labels represent expression in leaf and meristem tissues, respectively. Direction arrows represent activation with $\gamma$ (Hill) functions, where squares describe the dynamic variables, blocked ones inhibition with $\kappa$ functions and parallelograms describe the input variables. Dashed arrows show the delayed transport and action of $\textit{FT}$ onto $\textit{AP}1$ and $\textit{SOC}1$. Junction symbol next to $\textit{AP}1$ shows the multiple interactions from $\textit{LFY}$ to $\textit{AP}1$ (Color figure online)

The deterministic model proposed in Valentim et al. (2015) is represented schematically in Fig. 1. Here, the transcription of $\textit{FT}$ is controlled by *SVP* and *FLC* in the leaves as shown in Fig. 1. After $\textit{FT}$ is created in the leaves, it transfers to the meristem to interact with $\textit{FD}$. They activate the $\textit{SOC}1$ expression together and $\textit{AP}1$ individually (Valentim et al. 2015; Wang et al. 2014). $\textit{SOC}1$ is activated by *FT* / *FD*, *AGL*24 and itself. Moreover, the expression of $\textit{SOC}1$ is repressed by *SVP* and *FLC* in the meristem. $\textit{LFY}$ is assumed to move through a positive feedback loop with the dimerisation of *AGL*24 and $\textit{SOC}1$. $\textit{LFY}$ is also a positive regulator of $\textit{FD}$ and $\textit{AP}1$. The flowering process is determined by a direct positive input interaction among $\textit{LFY}$ and $\textit{AP}1$. When the $\textit{AP}1$ expression is started, the transcription variable $\textit{AP}1$ arranges the floral transition by identifying the status of floral meristem and regulating the gene expressions comprised in flower progress (Valentim et al. 2015; Kaufmann et al. 2010). Following Valentim et al. (2015), protein and RNA levels are assumed to be linearly correlated with each other. $SVP_{l}$ and $FLC_{l}$ represent the gene expression of *SVP* and *FLC* in the leaves and $SVP_{m}$ and $FLC_{m}$ in the meristem. These four components, $SVP_{l}$, $FLC_{l}$, $SVP_{m}$ and $FLC_{m}$, are independent input variables for the system which are linearly interpolated from the experimental data.

These considerations led to the following system of six differential equations with one delay (Valentim et al. 2015),

1 $$\begin{aligned} \dot{x}_1= & {} \beta _{1}\gamma _{1}^n(x_2) + \beta _{2}\gamma _{2}(x_4) + \beta _{3}\gamma _{3}(x_6(\tau )) - d_1x_1, \nonumber \\ \dot{x}_2= & {} \beta _{4}\gamma _{4}(x_1) + \beta _{5}\gamma _{5}(x_3) + \beta _{6}\gamma _{6}(x_5) - d_2x_2, \nonumber \\ \dot{x}_3= & {} \left[ \beta _{7}\gamma _{7}(x_3) + \beta _{8}\gamma _{8}(x_5) + \beta _{9}\gamma _{9}(x_4)\gamma _{10}(x_6(\tau ))\right] \kappa _{11}(x_7)\kappa _{12}(x_8) - d_3x_3, \nonumber \\ \dot{x}_4= & {} \beta _{10}\gamma _{13}(x_2) - d_4x_4, \nonumber \\ \dot{x}_5= & {} \beta _{11}\gamma _{14}(x_3) - d_5x_5, \nonumber \\ \dot{x}_6= & {} \beta _{12}\kappa _{15}(x_9)\kappa _{16}(x_{10}) - d_6x_6, \end{aligned}$$

where the functions are defined as

$$\begin{aligned}&\gamma _{j}^n(x_{i})=\dfrac{x_{i}^n}{x_{i}^n + K_{j}^n} , \ \ \gamma _{j}(x_{i})=\gamma _{j}^1(x_{i}) \quad \hbox { and }\quad \kappa _{j}(x_{i})=\dfrac{K_{j}}{x_{i} + K_{j}}, \\&\qquad (j=1,\ldots ,16 \hbox { and } i=1,\ldots ,10). \end{aligned}$$

In system (1), the variables ${x}_i$ are protein concentrations, which depend on time *t*, and represent the genes as follows:

$$\begin{aligned}&AP1 \rightarrow x_{1}, \ LFY \rightarrow x_{2}, \ SOC 1 \rightarrow x_{3}, \ FD \rightarrow x_{4}, \ AGL 24 \rightarrow x_{5}, \ FT \rightarrow x_{6}, \\&SVP_{m} \ \rightarrow x_{7}, \ FLC_{m} \rightarrow x_{8}, \ SVP_{l} \rightarrow x_{9} \hbox { and } FLC_{l} \rightarrow x_{10}. \end{aligned}$$

The delayed time $\tau =t-\Delta$ appears in the equations for ${x}_1$ and ${x}_3$. The reason for this is that $\textit{FT}$ occurs in the leaves and then moves to the meristem with some time delay $\Delta$, which is assumed to take less than 24 h (Valentim et al. 2015). The Hill functions $\gamma _{j}$ and $\kappa _j$ represent activations inhibition kinetics, respectively. The coefficient *n* of the Hill function $\gamma _{1}$ represents the cooperativity in the regulation of $\textit{AP}1$ by $\textit{LFY}$ and is assumed to be a positive integer. The meaning of the other coefficients is provided in Table 1. Their values, estimated from experimental data using polynomial data fitting in Valentim et al. (2015), are given in Table 2.

**Table 1:** Description and range for the parameters in the dynamic model

Parameters	Description	Range
$\beta _i$, $(i=1,2,\ldots , 12)$	Maximum transcription rate	$[0.001, 200]\,\hbox {nM}*\hbox {min}^{-1}$
$K_i$, $(i=1,2,\ldots , 16)$	Abundance at half maximum transcription rate	[0.001, 2000] nM
$d_i$, $(i=1,2,\ldots , 6)$	Gene products degradation rate	$[0.001, 1]\,\hbox {min}^{-1}$
$\Delta$	Delay	[0, 1] days

**Table 2:** Model parameters, estimated from experimental gene expression data using a polynomial fit (Valentim et al. 2015)

Parameters	Estimated values	Parameters	Estimated values (nM)	Parameters	Estimated values
$\beta _{1}$	$99.8\,\hbox {nM}\times \hbox {min}^{-1}$	$K_{1}$	9.82	$K_{13}$	7.9 nM
$\beta _{2}$	$5\,\hbox {nM}\times \hbox {min}^{-1}$	$K_{2}$	700	$K_{14}$	125 nM
$\beta _{3}$	$10\,\hbox {nM}\times \hbox {min}^{-1}$	$K_{3}$	10.1	$K_{15}$	0.63 nM
$\beta _{4}$	$22\,\hbox {nM}\times \hbox {min}^{-1}$	$K_{4}$	346	$K_{16}$	985 nM
$\beta _{5}$	$2.4\,\hbox {nM}\times \hbox {min}^{-1}$	$K_{5}$	842	$d_{1}$	$0.86\,\hbox {min}^{-1}$
$\beta _{6}$	$0.79\,\hbox {nM}\times \hbox {min}^{-1}$	$K_{6}$	1011	$d_{2}$	$0.017\,\hbox {min}^{-1}$
$\beta _{7}$	$64\,\hbox {nM}\times \hbox {min}^{-1}$	$K_{7}$	695	$d_{3}$	$0.11\,\hbox {min}^{-1}$
$\beta _{8}$	$0.52\,\hbox {nM}\times \hbox {min}^{-1}$	$K_{8}$	1182	$d_{4}$	$0.0075\,\hbox {min}^{-1}$
$\beta _{9}$	$189\,\hbox {nM}\times \hbox {min}^{-1}$	$K_{9}$	2.4	$d_{5}$	$0.001\,\hbox {min}^{-1}$
$\beta _{10}$	$8.5\,\hbox {nM}\times \hbox {min}^{-1}$	$K_{10}$	4.8	$d_{6}$	$0.1\,\hbox {min}^{-1}$
$\beta _{11}$	$100\,\hbox {nM}\times \hbox {min}^{-1}$	$K_{11}$	909	$\Delta$	0.5 day
$\beta _{12}$	$51\,\hbox {nM}\times \hbox {min}^{-1}$	$K_{12}$	501		

**Fig. 2:**
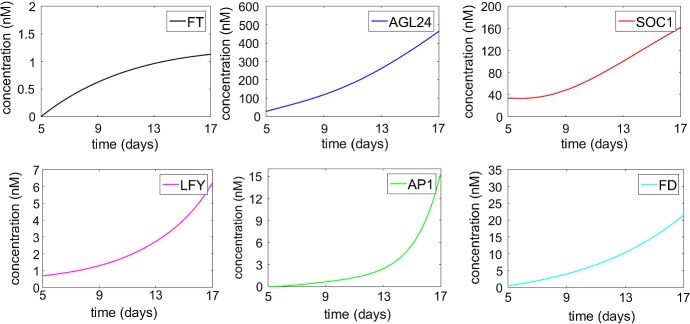
Numerical simulation of system (1) after germination. The initial values for $\textit{FT}$, *AGL*24, $\textit{SOC}1$, $\textit{LFY}$, $\textit{AP}1$ and $\textit{FD}$ are taken from Valentim et al. (2015) as 0.00056 nM, 27.69 nM, 33.3 nM, 0.68 nM, 0.00056 nM and 0.431 nM, respectively (Color Figure Online)

The behaviour of system (1) is simulated in Fig. 2 using the parameters in Table 2 and the experimental data used in Valentim et al. (2015). The initial conditions are taken from the experimental data. The sharp rise in $\textit{AP}1$ from 13 to 17 days after germination can be interpreted as a predictor of flowering.

As is known from laboratory experiments (see Krämer 2015), *Arabidopsis thaliana* is an annual plant and its flowering is limited to approximately two to four weeks after germination. In this context, a non-trivial stable steady state can be seen as an attracting point for the flowering process. Hence, in the next section, we turn to the analysis of the steady state of the flowering model to determine its behaviour, give conditions on its initiation and investigate the terminal stages of the flowering process.

### Steady State and Stability Analysis of the Deterministic Model

Steady states of the system represent equilibrium points about which the dynamics can be studied using linear stability analysis. It helps to describe the behaviour of a delayed system solution by considering the trajectories in a phase space of all dependent variables. As mentioned previously, we interpret a stable steady state as an attractor for the flowering process. Therefore, if the Arabidopsis flowering is successful, then there exists at least one strictly positive stable steady state.

Hence, for DDEs of form (1), which can be presented as

2 $$\begin{aligned} \dfrac{\mathrm{d}x_{i}}{\mathrm{d}t}=f_{i}(x_{1},x_{2},\ldots ,x_{5},x_{6},x_{6}(\tau )), \quad i=1,2,\ldots ,6, \end{aligned}$$

the equilibrium points ${\bar{x}}=({\bar{x}}_1,{\bar{x}}_2,\ldots ,{\bar{x}}_{5},{\bar{x}}_{6})$ can be found by considering the equations $f_{i}({\bar{x}}_{1},{\bar{x}}_{2},\ldots ,{\bar{x}}_{5},{\bar{x}}_{6},{\bar{x}}_{6}) = 0$, at ${\bar{x}}_{6}(\tau )={\bar{x}}_{6}$. In our further consideration, we assume that the independent input variables $x_{7},\ldots ,x_{10}$ in system (1) are constant and equal to their initial values as given in Table 4 in “Appendix”, to derive the steady states. This results into

3a $$\begin{aligned} {\bar{x}}_{1}= & {} \dfrac{\beta _{1}\gamma _{1}^n({\bar{x}}_{2}) + \beta _{2}\gamma _{2}({\bar{x}}_{4}) + \beta _{3}\gamma _{3}({\bar{x}}_6)}{d_{1}}, \end{aligned}$$

3b $$\begin{aligned} {\bar{x}}_{2}= & {} \dfrac{\beta _{4}\gamma _{4}({\bar{x}}_1) + \beta _{5}\gamma _{5}({\bar{x}}_3) + \beta _{6}\gamma _{6}({\bar{x}}_5)}{d_{2}}, \end{aligned}$$

3c $$\begin{aligned} {\bar{x}}_{3}= & {} \dfrac{[\beta _{7}\gamma _{7}({\bar{x}}_3) + \beta _{8}\gamma _{8}({\bar{x}}_5) + \beta _{9}\gamma _{9}({\bar{x}}_4)\gamma _{10}({\bar{x}}_6)] \times \kappa _{11}(x_{7})\kappa _{12}(x_{8})}{d_{3}}, \end{aligned}$$

3d $$\begin{aligned} {\bar{x}}_{4}= & {} \dfrac{\beta _{10}\gamma _{13}({\bar{x}}_2)}{d_{4}}, \end{aligned}$$

3e $$\begin{aligned} {\bar{x}}_{5}= & {} \dfrac{\beta _{11}\gamma _{14}({\bar{x}}_3)}{d_{5}}, \end{aligned}$$

3f $$\begin{aligned} {\bar{x}}_{6}= & {} \dfrac{\beta _{12}\kappa _{15}(x_{9})\kappa _{16}(x_{10})}{d_6} = u, \end{aligned}$$

where *u* is a constant. Here we focus on the case $n=3$, which is the value obtained by fitting experimental data in Valentim et al. (2015). It is easily seen that no trivial steady state is present whenever the constant inputs $x_9$ and $x_{10}$ are assumed to be nonzero. To find all equilibrium points using the assumption above, we follow the steps given in “Appendix” A.1. Eliminating ${\bar{x}}_2$ from (34) and (35), we obtain a 17th-degree polynomial equation for ${\bar{x}}_3$ which we do not reproduce here. Hence, it is seen that ${\bar{x}}_6$ is obtained directly from the input concentrations, while ${\bar{x}}_1$, ${\bar{x}}_2$, ${\bar{x}}_3$, ${\bar{x}}_4$ and ${\bar{x}}_5$ are nonlinearly linked with each other. Using values for estimated parameters (Table 2) and the independent input variables (Table 4 in “Appendix”), it can be seen numerically that there exists a unique positive steady state, as given in Table 3.

**Table 3:** Unique positive steady state for concentrations (in nm), obtained by using the parameters in Table 2 and initial values in Table 4, given in “Appendix”

${\bar{x}}_1$	${\bar{x}}_2$	${\bar{x}}_3$	${\bar{x}}_4$	${\bar{x}}_5$	${\bar{x}}_6$
121.567	452.395	827.835	1113.882	86,881.258	2.037

**Fig. 3:**
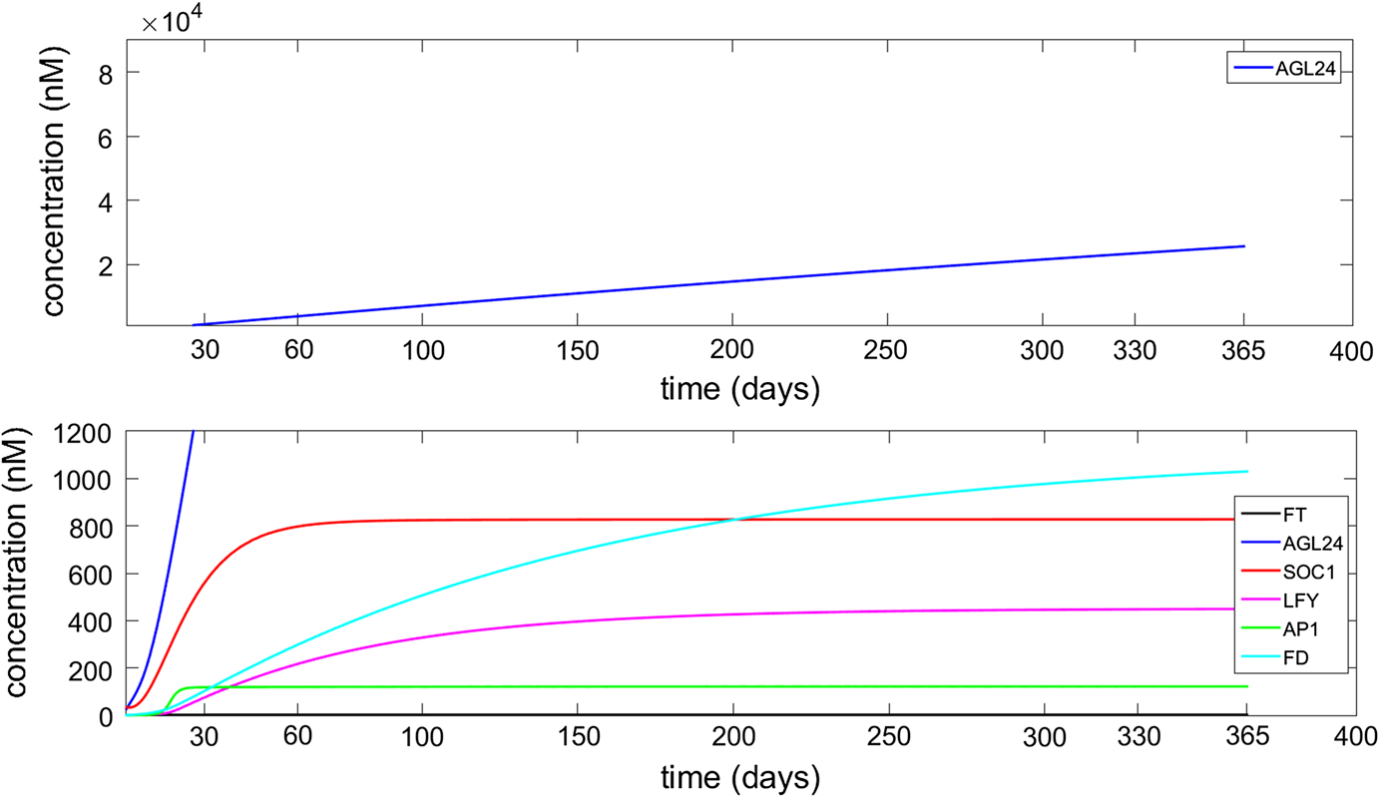
Numerical solution of the system showing the asymptotic stability of the steady state. The blue line representing *AGL*24 is split between two graphs as the values of concentration vary from 0 to 30,000. The initial values are as in Fig. 2 (Color figure online)

Numerical simulation of system (1), with MATLAB R2015b, showing convergence to the steady state, is presented in Fig. 3. The time for which $\textit{AP}1$ sees a sharp rise is in agreement with the time at which the most dramatic part of the flowering takes place, and the time for which $\textit{AP}1$ reaches its steady state is in agreement with the ending of flowering process, which has been observed between two to four weeks in laboratory experiments (Krämer 2015; Sanda et al. 1997; Valentim et al. 2015). Our simulations show that the main features of the system behaviour would not change for different values of the input variables, apart from a slight variation in the numerical values of the steady-state concentrations.

Linearisation of the nonlinear system (1) is required to analyse the local stability of this dynamic model at its steady states $({\bar{x}}_{1},{\bar{x}}_{2},{\bar{x}}_{3},{\bar{x}}_{4},{\bar{x}}_{5},{\bar{x}}_{6})$. Stability analysis is used to establish threshold conditions on the model parameters for the flowering of the plant. Therefore, we analyse the linear stability of the model in detail, and explicit conditions for local stability are formulated using the Routh–Hurwitz criterion. This gives the following theorem, for which further details can be seen in “Appendix A.2”.

#### Theorem 1

A steady state of the nonlinear system (1) is locally asymptotically stable iff all the roots of the polynomial

4 $$\begin{aligned} P_2(\lambda )= & {} \lambda ^5+a_1\lambda ^4+a_2\lambda ^3+a_3\lambda ^2+a_4\lambda +a_5, \end{aligned}$$

have negative real parts, that is iff the following conditions are satisfied,

$$\begin{aligned}&a_i>0, \ \ i=1, \ldots , 5, \\&a_1a_2a_3 + a_1a_5 \> \ a_3^2+a_1^2a_4, \ \ \hbox { and }\\&(a_1a_4-a_5)(a_1a_2a_3+a_1a_5-a_3^2-a_1^2a_4) \ > \ a_5(a_1a_2-a_3)^2, \end{aligned}$$

where

$$\begin{aligned}&a_1 = d_1+d_2+d_3+d_4+d_5-F ,\\&a_2 = -HL - d_5F + d_3d_5 + (d_3+d_5-F)(d_1+d_2+d_4)\\&\quad \qquad - AC + d_1d_2 + d_1d_4 + d_2d_4 , \\&a_3 = - (d_1+d_2+d_4)(HL+d_5F-d_3d_5)\\&\quad \qquad - (d_3+d_5-F)(AC-d_1d_2-d_1d_4-d_2d_4) \\&\quad \qquad - (d_4AC + BCK + DGK - d_1d_2d_4) ,\\&a_4 = (HL+d_5F-d_3d_5)(AC-d_1d_2-d_1d_4-d_2d_4) \\&\quad \qquad - (d_3+d_5-F)(d_4AC+BCK-d_1d_2d_4) - (EGKL+(d_1+d_5)DGK) ,\\&a_5 = (d_4AC+BCK-d_1d_2d_4)(HL+d_5F-d_3d_5) - (d_1d_5DGK+d_1EGKL) , \end{aligned}$$

and the quantities $A,B,C,\ldots$ are defined in “Appendix A.2”. Otherwise, the steady state of the system is unstable.

In summary, the conditions in Theorem 1 show that the local stability of system (1) at the steady state depends on values of parameters and concentrations. Given the high dimensionality of the parameter space, it is a difficult task to fully describe regions where stability holds. Nonetheless, it is worth noting that the delay $\tau$ does not influence stability in this particular system. No bifurcation has been numerically detected in the parameter ranges considered in this work.

To reduce the number of parameters, we now introduce a simpler system which reproduces the essential behaviour of system (1). Therefore, we performed local parameter sensitivity analysis to figure out the most important parameters in GRN (see “Appendix C”), which are $\beta _{1}$, $\beta _{4}$, $\beta _{5}$, $K_{1}$, $K_{4}$, $K_{5}$ and $d_1$, and all belong to the first two equations. For this purpose, we consider subsystems and analyse their stability to understand the behaviour of system (1).

## Deterministic Model of the Simplified Network

The complex large regulatory network represented in (1) can be simplified while still saving its core structure. By decoupling some concentrations, it is possible to reduce the number of differential equations of the large system. One can see from the analysis in the previous section that the main contribution to the dynamics is from protein concentrations related to $\textit{AP}1$, $\textit{LFY}$ and $\textit{SOC}1$. Indeed, from the structure of system (1), it is seen that $x_4$, $x_5$ and $x_6$ can be computed explicitly from the knowledge of $x_2$, $x_3$ and the external outputs. Hence, we focus the analysis on these genes to investigate how they contribute to the regulation of $\textit{AP}1$.

Hence, by considering $\dot{x}_{i}(t)=0$ for $i=4,5$ and 6, we obtain the following system of differential equations for the variables $x_1$, $x_2$ and $x_3$:

5 $$\begin{aligned} \dot{x}_{1}= & {} \dfrac{V_1x_{2}^3}{x_{2}^3+S_1^{3}} + \dfrac{V_2x_2}{S_2x_2+S_3} + U_1 - d_{1}x_{1}, \nonumber \\ \dot{x}_{2}= & {} \dfrac{V_3x_1}{x_1+S_4} + \dfrac{V_4x_3}{x_3+S_5} + \dfrac{V_5x_3}{S_6x_3+S_7} - d_{2}x_{2}, \nonumber \\ \dot{x}_{3}= & {} \dfrac{U_{2}V_6x_2}{S_8x_2+S_9} + \dfrac{V_7x_3}{x_3+S_{10}} + \dfrac{V_8x_3}{S_{11}x_3+S_{12}} - d_{3}x_{3}, \end{aligned}$$

where the parameters are defined by

$$\begin{aligned}&V_1=\beta _{1}, \ V_2=\beta _{2}\beta _{10}, \ V_3=\beta _{4}, \ V_4=\beta _{5}, \ V_5=\beta _{6}\beta _{11}, \ V_6=\beta _{9}\beta _{10}\kappa _{11}\kappa _{12}, \\&V_7=\beta _{7}\kappa _{11}\kappa _{12}, \ V_8=\beta _{8}\beta _{11}\kappa _{11}\kappa _{12}, \ S_1=K_1, \ S_2=\beta _{10}+d_4K_2, \ S_3=d_4K_2K_{13}, \\&S_4=K_4, \ S_5=K_5, \ S_6=\beta _{11}+d_5K_6, \ S_7=d_5K_6K_{14}, \ S_8=\beta _{10}+d_4K_9, \\&S_9=d_4K_9K_{13}, \ S_{10}=K_7, \ S_{11}=\beta _{11}+d_5K_8, \ S_{12}=d_5K_8K_{14},\\&U_1=\beta _3u(u+K_3)^{-1}, U_2=u({u+K_{10}})^{-1}. \end{aligned}$$

The constant *u* defined in (3f) represents the constant value of the $\textit{FT}$ concentration, while $U_1$ and $U_2$ determine the effect of $\textit{FT}$ and $\textit{FT}$-$\textit{FD}$ combination on $\textit{AP}1$ and $\textit{SOC}1$, respectively.

**Fig. 4:**
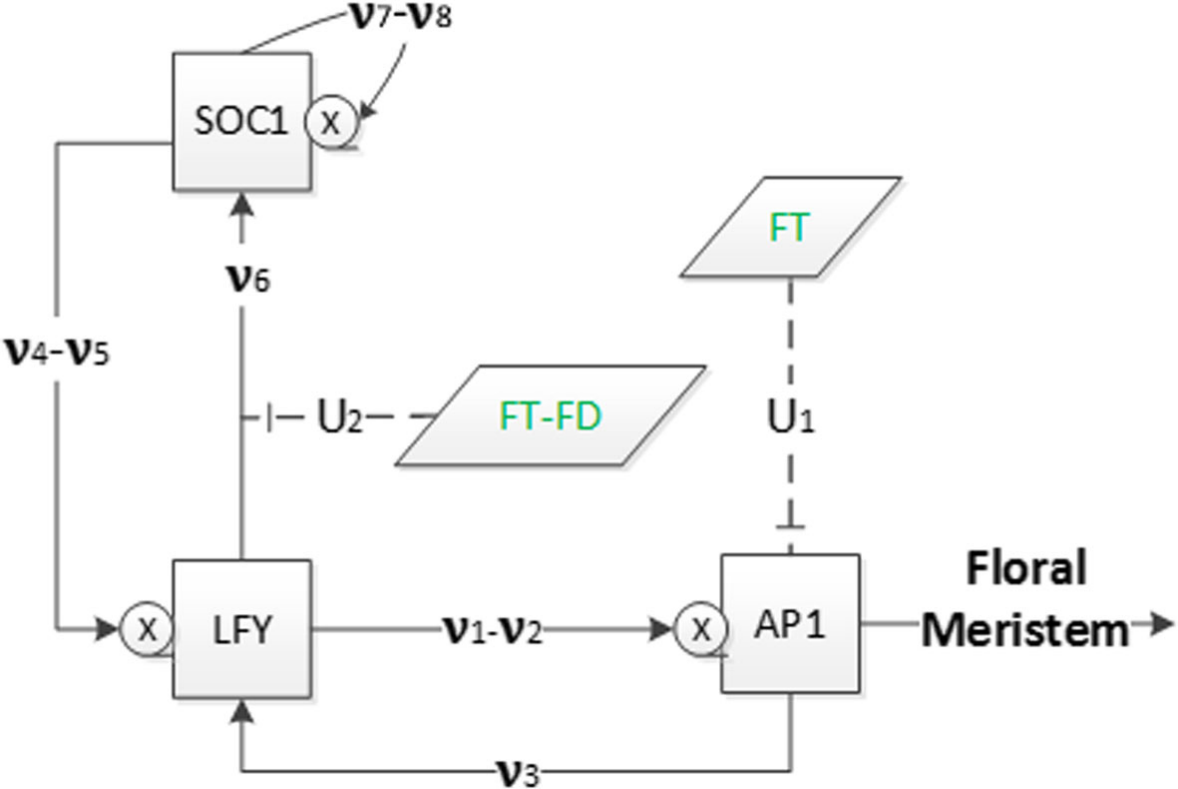
Flowchart of system (5). The meaning of symbols is the same as in Fig. 1

The network of system (5) is described in Fig. 4. The difference with the model in Fig. 1 is that *AGL*24 is not involved with the external input variables *SVP* and *FLC* as they are decoupled. This network consists of three internal state variables representing $\textit{SOC}1$, $\textit{LFY}$ and $\textit{AP}1$, which determine the main dynamics of system (5), and two external input variables $\textit{FT}$ and $\textit{FT}$-$\textit{FD}$ combination which are constant.

**Fig. 5:**
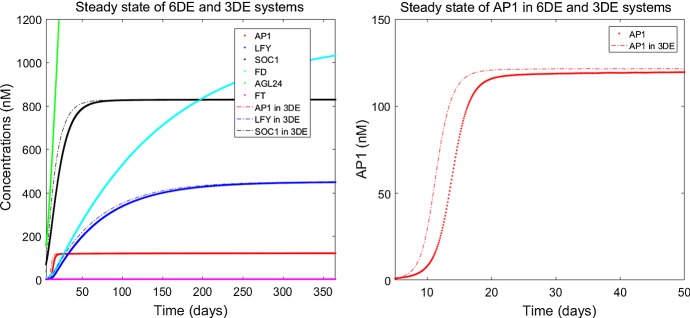
Figure on the left shows a 1-year comparison of the numerical solutions for systems (1) (with solid lines) where only $\textit{FT}$ is constant and (5) (with dashed lines) after decoupling *AGL*24 and $\textit{FD}$ which are assumed constant alongside $\textit{FT}$. Figure on the right represents comparison of $\textit{AP}1$ in both systems for 50 days. The initial conditions are as in Fig. 2 (Color Figure Online)

The numerical solution of the non-decoupled variables $\textit{SOC}1$, $\textit{LFY}$ and $\textit{AP}1$ in system (5) is compared with the numerical solution of system (1) in Fig. 5. The convergence of $x_1$ is affected by the constant values used for $x_4, x_5, x_6$. Using the steady-state values, which represent the highest concentrations for these variables, leads to a slightly faster converging graph for $\textit{AP}1$ that can be seen in Fig. 5 on the right. This result shows that decoupling some concentrations on the system can still capture the essential behaviour of the complex network for these non-constant variables.

Linear stability of the simplified model and explicit conditions for local stability at its steady states (${\bar{x}}_1$, ${\bar{x}}_2$, ${\bar{x}}_3$) are formulated using the Routh–Hurwitz criterion in Theorem 2, and further details can be seen in “Appendix A.3”.

### Theorem 2

A steady state of the nonlinear simplified system (5) is locally asymptotically stable iff all the roots of the polynomial

6 $$\begin{aligned} P(\lambda )= & {} \lambda ^3+a_1\lambda ^2+a_2\lambda +a_3, \end{aligned}$$

have negative real parts, that is iff the conditions, $a_i>0, \ \ i=1, \ldots ,3$ and $a_1a_2>a_3$, are satisfied, where

$$\begin{aligned}&a_1=d_1+d_2+d_3-{\mathcal {E}}, \ \ \ a_2=d_1d_2+d_1d_3+d_2d_3-(d_1+d_2){\mathcal {E}}-{\mathcal {A}}{\mathcal {B}}-{\mathcal {C}}{\mathcal {D}},\\&\quad a_3=d_1d_2d_3 + {\mathcal {A}}{\mathcal {B}}{\mathcal {E}} - d_1d_2{\mathcal {E}} - d_1{\mathcal {C}}{\mathcal {D}} - d_3{\mathcal {A}}{\mathcal {B}}, \end{aligned}$$

and the quantities ${\mathcal {A}}, {\mathcal {B}}, \ldots$ are defined in “Appendix A.3”. Otherwise, the steady state of the simplified system is unstable.

## Deterministic Models of Motifs

To further reduce the complexity of system (1), we use the approach in Pullen et al. (2013) and reduce system (5) from three to two equations to understand the essential characteristics of the floral transition by considering the two components, $\textit{LFY}$ and $\textit{AP}1$, which constitute the minimal set for enabling the transition to floral meristem (Mandel et al. 1992). Here, we model minimal regulatory networks of core components consisting of the protein concentrations for $\textit{LFY}$, $\textit{AP}1$, $\textit{FT}$ and $\textit{FD}$. We consider the simplified subsystem proposed in Figure 1(b) in Pullen et al. (2013) to establish the essential characteristics of the floral transition. From system (5), one can integrate the third equation to obtain $x_3$ in terms of $x_1$ and $x_2$ and the various constant inputs, which also depend on $\textit{FT}$ and $\textit{FD}$. The simplified system is represented in Fig. 4 and results from considering constant $\textit{SOC}1$ concentrations ($\dot{x}_3 = 0$). The reason we use these four genes is: $\textit{AP}1$ and $\textit{LFY}$ are key floral meristem identity genes in the network of Arabidopsis flowering (Irish 2010; Wellmer and Riechmann 2010) and $\textit{FT}$ induces flowering through the activation of these two genes in a feed-forward circuit (Kardailsky et al. 1999) where $\textit{FD}$ has a significant role for $\textit{FT}$ signalling.

**Fig. 6:**
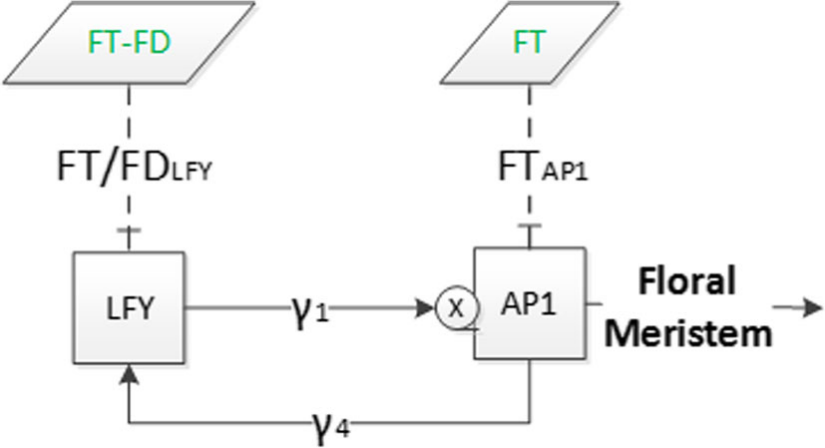
Flowchart of the simplified system. Meaning of symbols is as in previous diagrams

$\textit{AP}1$ and $\textit{LFY}$ activate each other in the integration of flowering signals where they are mutual transcriptional activators (Liljegren et al. 1999). As these concentrations are key floral meristem identity genes in the network, the subsystem is based on these two genes and take into account the importance of the network activators and inhibitors. Additionally, we incorporate the action of $\textit{FT}$-$\textit{FD}$ as a combined activator/inhibitor, as suggested in Wang et al. (2014) and Pullen et al. (2013). Ignoring the change in $\textit{SOC}1$ concentration in the network in Fig. 4, we can redefine the simplified network as shown in Fig. 6, where $\textit{LFY}$ and $\textit{AP}1$ represent the main dynamics of this system, and $\textit{FT}$-$\textit{FD}$ and $\textit{FT}$ are the external input variables.

The analysis of the subsystem in Fig. 6 allows to investigate the activation and inhibition processes and provides ranges for input parameters which lead to the existence of stable solutions. Here, the effect of $\textit{FT}$-$\textit{FD}$ and $\textit{FT}$ is described by $F_1$ for $\textit{LFY}$ and $F_2$ for $\textit{AP}1$ in the system.

7 $$\begin{aligned} \dfrac{dx_1}{dt}= & {} \beta _{1}\left( \dfrac{{x_2}^n}{{x_2}^n+{K_1}^n}\right) F_2-d_1x_1, \nonumber \\ \dfrac{dx_2}{dt}= & {} \beta _{4}\left( \dfrac{x_1}{x_1+K_4}\right) F_1-d_2x_2. \end{aligned}$$

**Fig. 7:**
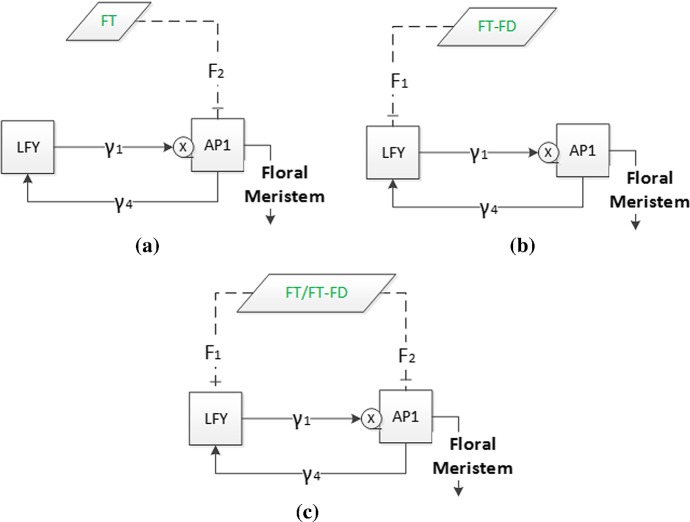
Effect of $\textit{FT}$ and combined effect of $\textit{FT}$-$\textit{FD}$ inhibitor/activator actions on $\textit{AP}1$ and $\textit{LFY}$. Squares describe the floral meristem identity genes, LFY and AP1, which are the dynamic variables, and parallelograms describe the combination of external input variables, $\textit{FT}$ and $\textit{FT}$-$\textit{FD}$, which are repressor/activator of $\textit{AP}1$ and $\textit{LFY}$. Junction symbol next to AP1 shows the multiple interactions from $\textit{AP}1$ and $\textit{LFY}$, and $\gamma _{1}$ and $\gamma _{4}$ (Hill) functions are as in system (1). **a** Subsystem 1, the action is on $\textit{AP}1$ only, **b** subsystem 2, the action is on $\textit{LFY}$ only, and **c** subsystem 3, the action is on both $\textit{AP}1$ and $\textit{LFY}$. **a** Subsystem 1, $F_1=1$, $F_2\ne 1$. **b** Subsystem 2, $F_2=1$, $F_1\ne 1$. **c** Subsystem 3, $(F_1$ and $F_2)\ne 1$

Here, $F_1$ and $F_2$ are joint inhibiting (when $F_i<1$) and activating (when $F_i>1$) constants, $i=1,2$. The variables $x_1$ and $x_2$ represent $\textit{AP}1$ and $\textit{LFY}$, respectively, as defined before, and we select the parameters $\beta _1$, $\beta _4$, $K_{1}$, $K_{4}$, $d_1$ and $d_2$ to be the same as previously (Table 2). This assumption relies on the statistical importance of these parameters, as determined in the sensitivity analysis. We analyse subsystem (7) in three cases including different $\textit{AP}1$-$\textit{LFY}$ activation pathways. The first one shows the inhibition and activation of $\textit{FT}$ effect on $\textit{AP}1$ while $F_1=1$; the second one, $\textit{FT}$-$\textit{FD}$ effect on $\textit{LFY}$ while $F_2=1$. The third case shows the equal inhibition or activation effect of $\textit{FT}$-$\textit{FD}$ and $\textit{FT}$ on $\textit{LFY}$ and $\textit{AP}1$ ($F_1=F_2$), respectively. The three realisations of the $\textit{FT}$-$\textit{FD}$ and $\textit{FT}$ actions are given in Fig. 7.

The aim of the first and second subsystems is to analyse the effect of input variables on $\textit{AP}1$ and $\textit{LFY}$, individually. The third subsystem is aimed to obtain the effect of input variables when they have an equal action on both main concentrations. The parameters in Table 2 are used to investigate the behaviour of the input variables whether they play an inhibitor or an activator role.

### Steady States of Motifs

The steady states $({\bar{x}}_1,{\bar{x}}_2)$ of system (7) are found by considering the right-hand side of the equations equal to zero:

8 $$\begin{aligned} \beta _{1}\left( \dfrac{{\bar{x}}_2^n}{{\bar{x}}_2^n+K_{1}^n}\right) F_2-d_1{\bar{x}}_1 = 0, \ \ \ \ \ \ \ \beta _{4}\left( \dfrac{{\bar{x}}_1}{{\bar{x}}_1+K_{4}}\right) F_1-d_2{\bar{x}}_2 = 0. \end{aligned}$$

Here, it is easily shown that the trivial solution $({\bar{x}}_1,{\bar{x}}_2)=(0,0)$ is a stable steady state of system (7). Although gene concentrations cannot formally be zero, the trivial steady state corresponds to a state where only small quantities are present due to non-modelled or stochastic effects. Hence, we now focus on the non-trivial positive steady states, which can be obtained through the following process. Eliminating ${\bar{x}}_1$ from the first two equations in (8), we have

9 $$\begin{aligned} \dfrac{\beta _{1}F_2}{d_1}\left( \dfrac{{\bar{x}}_2^n}{{\bar{x}}_2^n+{K_{1}}^n}\right) = \dfrac{d_2K_{4}{\bar{x}}_2}{\left( \beta _{4}F_1-d_2{\bar{x}}_2\right) }, \end{aligned}$$

where $\beta _{4}F_1-d_2{\bar{x}}_2 > 0$ as we only consider positive concentrations. This gives an upper bound for existence of ${\bar{x}}_2$ for all parameter values,

10 $$\begin{aligned} {\bar{x}}_2 < \dfrac{\beta _{4}F_1}{d_2}. \end{aligned}$$

Rearranging (9) implies that any non-trivial steady state for ${\bar{x}}_2$ satisfies the polynomial equation

11 $$\begin{aligned} (\omega _1+\omega _2F_2){\bar{x}}_2^n-\omega _3F_1F_2{\bar{x}}_2^{n-1}+\omega _4=0, \end{aligned}$$

where $\omega _1=d_1d_2K_{4}$, $\omega _2=d_2\beta _{1}$, $\omega _3=\beta _{1}\beta _{4}$ and $\omega _4=d_1d_2K_{1}^nK_{4}$. Focusing only on positive solutions, Descartes’ rule of signs indicates that (11) possesses either zero or two real positive roots, while others are complex or negative. As a consequence, system (7) has either zero or two positive steady states. We will analyse the conditions for positive real roots in further sections by using the parameter values in Table 2.

### Deterministic Stability of Motifs

The dynamical subsystem (7) must have at least one stable steady state to represent the behaviour of the Arabidopsis flowering. In order to determine whether the positive equilibrium points (${\bar{x}}_1, {\bar{x}}_2$) are locally stable, we compute the eigenvalues of the Jacobian matrix evaluated at the equilibrium points. The Jacobian matrix of systems (7) is given as,

12 $$\begin{aligned} J_{({{\bar{x}}_1},{{\bar{x}}_2})}=\left[ \begin{array}{cc} -d_{1} &{} \dfrac{n\beta _{1}K_{1}^nF_2{\bar{x}}_2^{n-1}}{({{\bar{x}}_2^n+K_{1}^n})^2} \\ \dfrac{\beta _{4}K_{4}F_1}{{({{\bar{x}}_1+K_{4}})}^2} &{} -d_{2} \end{array}\right] . \end{aligned}$$

Requiring that $J_{({\bar{x}}_1, {\bar{x}}_2)}$ is Hurwitz stable leads to the necessary and sufficient stability condition

13 $$\begin{aligned} {\bar{x}}_2^{n-1}>\dfrac{nd_1d_2K_{1}^nK_{4}}{\beta _1\beta _4F_1F_2}. \end{aligned}$$

Combined with inequality (10), a given steady-state point ${\bar{x}}_2$ must satisfy

$$\begin{aligned} {\bar{x}}_2<\dfrac{\beta _4F_1}{d_2} \quad \text {and} \quad {\bar{x}}_2^{n-1}>\dfrac{nd_1d_2K_{1}^nK_{4}}{\beta _1\beta _4F_1F_2}, \end{aligned}$$

in order to be stable. Full details can be seen in “Appendix (A.4)”. The significance of this result is that the stability range is obtained in terms of the parameters of the system and the Hill coefficient *n*.

### Numerical Results for Deterministic Steady States and Stability of the Motifs

**Fig. 8:**
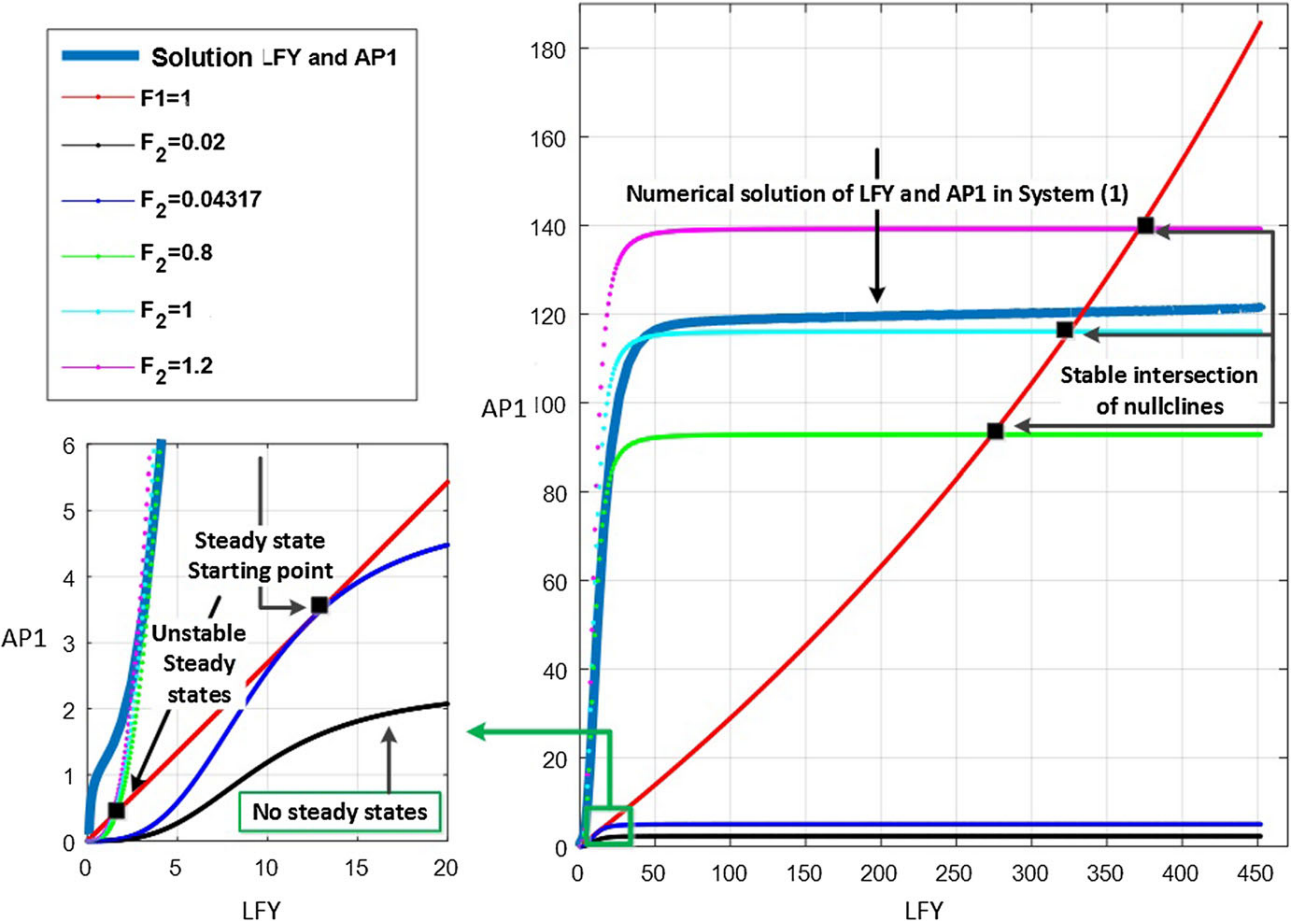
Nullclines (9) for subsystem 1 ($F_1=1$) with different values of $F_2$. The red curve represents LHS of (9); the other colours represent the RHS of (9). Intersections between the red curve and other curves correspond to steady states. The thick light blue line on the right of the figure represents the numerical solution of $\textit{LFY}$ and $\textit{AP}1$ from the original system (1) (Color figure online)

**Fig. 9:**
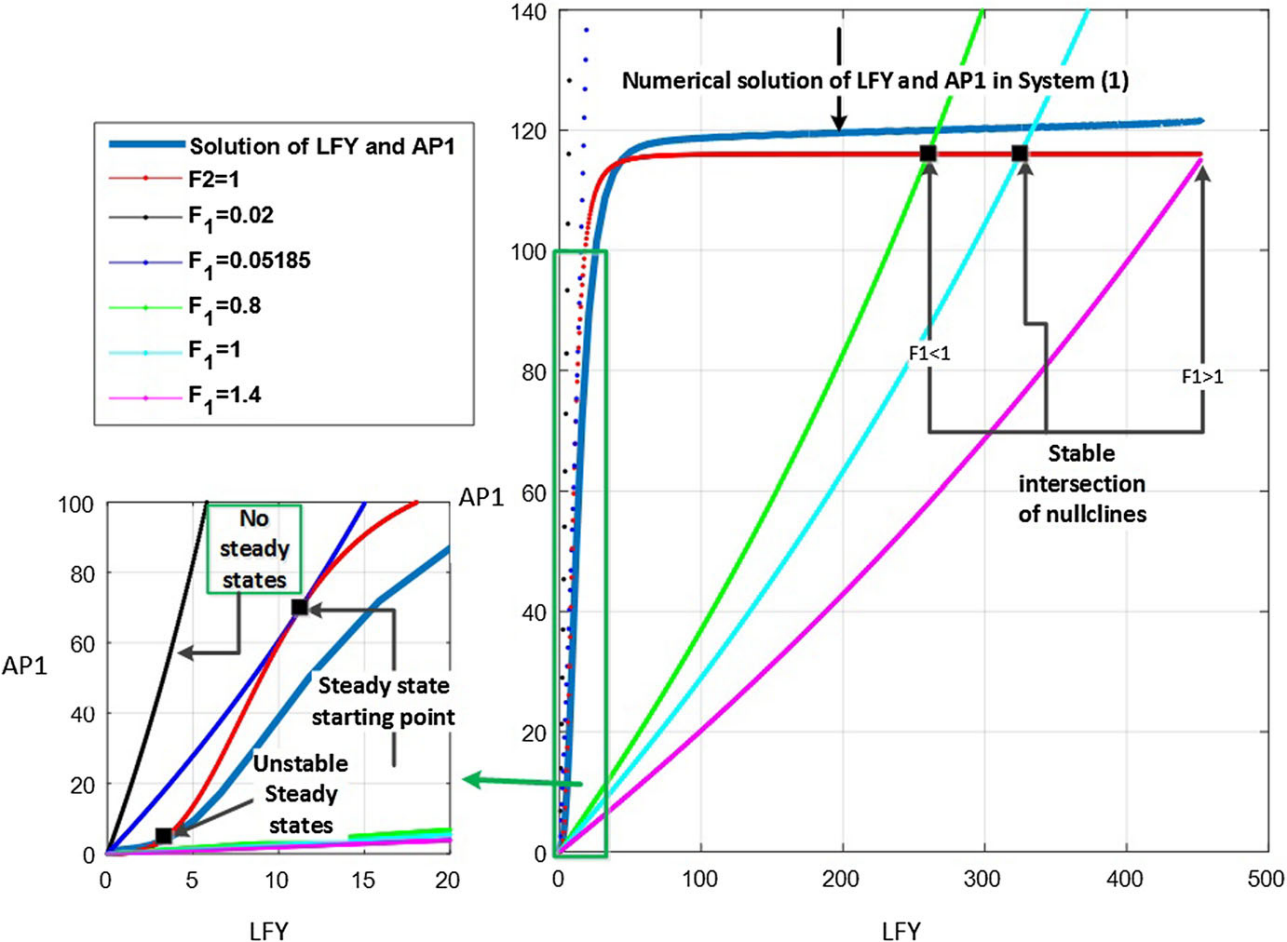
Nullclines (9) for subsystem 2 ($F_2=1$). The red curve represents RHS of (9); the other colours represent the LHS of (9). Intersections between red curve and other curves correspond to steady states. The thick light blue line on the right of the figure represents the numerical solution of $\textit{LFY}$ and $\textit{AP}1$ from the original system (1) (Color figure online)

Steady states are explicitly important because they offer vital knowledge on the flowering state. They can be identified by the intersection of nullclines obtained from equations (8), leading to equation (9). They are plotted in $({\bar{x}}_1,{\bar{x}}_2)$ space for the parameters in Table 2 and $n=3$. The results for subsystems 1 and 2 are shown in Figs. 8 and 9. In the graphs, reference points in the plane represent the values of $\textit{AP}1$ and $\textit{LFY}$ for specific interactions. Points where the nullclines intersect represent the steady states of the system. Non-intersecting nullclines indicate that there is no single steady state for system (7) for the given values of $F_1$ and $F_2$.

Let us now examine the stability of steady states of $\textit{LFY}$ and $\textit{AP}1$ for the case $n=3$, for which equation (11) becomes

14 $$\begin{aligned} (\omega _1+\omega _2 F_2) {\bar{x}}_2^3-\omega _3 F_1 F_2 {\bar{x}}_2^2 + \omega _4=0. \end{aligned}$$

Remembering that all coefficients $\omega _i, F_j$ are strictly positive, it is readily seen from Vieta’s formulae that equation (14) always possesses a negative root along with either two strictly positive or complex roots. Therefore, to obtain strictly positive roots, the discriminant $\Delta _3$ of the cubic (14) must be positive

15 $$\begin{aligned} \Delta _3= & {} \omega _4 (4(\omega _3F_1F_2)^3 - 27(\omega _1+\omega _2F_2)^2\omega _4) \ge 0. \end{aligned}$$

**Fig. 10:**
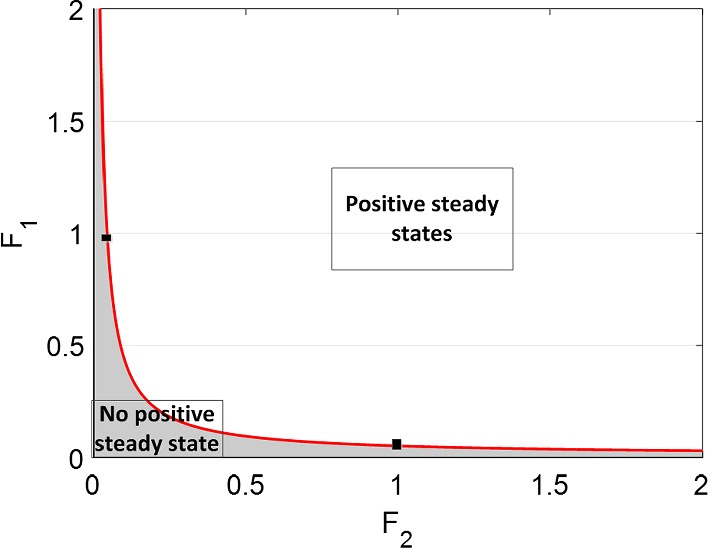
Minimum condition for the existence of positive steady states in the simplified system (7)

As values of $\omega _i$, $i=1,..,4$, can be calculated from the parameters in Table 2, the discriminant only depends on the unknown values of the external input variables $F_i$, ($i=1,2$) which represent the inhibiting ($F_i<1$) or activating ($F_i>1$) action of *FT* / *FD*. From the minimum condition of discriminant ($\Delta _3=0$), we find the critical values of $F_i$ for the existence of such roots. The plot in the ($F_1, F_2$) space given in Fig. 10 shows the region for the existence of positive steady states, delimited by the degeneracy condition $\Delta _3 = 0$ which gives rise to double roots.

**Subsystem 1** ($F_1=1$). Figure 8 shows the presence of a double root at $F_2 = 0.0431$ from which two distinct strictly positive equilibria emanate for $0.04317 < F_2 \le max\{F_2\}$. Hence, when no action of $\textit{FT}$-$\textit{FD}$ on $\textit{LFY}$ is present, the inhibition of $\textit{FT}$ on $\textit{AP}1$ starts at the value of $F_2=0.04317$ and activation can be seen for $F_2 >1$. Moreover, the behaviour of subsystem 1 is similar to system (1) for $F_2 \ge 0.04317$. The best match with the numerical solution of system (1) occurs for $F_2$ just above 1, with a best match of the steady-state value at $F_2 = 1.0476$. This in turn indicates an activation action of $\textit{FT}$ on $\textit{AP}1$.

**Subsystem 2** ($F_2=1$). A similar situation is seen in this case (Fig. 9). The numerical result for this subsystem indicates that in the absence of action of $\textit{FT}$ on $\textit{AP}1$, the inhibition of $\textit{FT}$-$\textit{FD}$ on $\textit{LFY}$ starts at the double root $F_1 = 0.05185$, from which it originates one stable and one unstable positive steady states. The behaviour of subsystem 2 is similar to system (1) for $F_1 \ge 0.05185$, while the best match with the numerical solution of system (1) can be seen in the activation of $\textit{FT}$-$\textit{FD}$ on $\textit{AP}1$ for $F_1$ just above 1, with a best match of the steady-state value at $F_1 = 1.3445$. In view of such information, we use $F_1$ and $F_2$ external input variables as an activator of the $\textit{LFY}$ and $\textit{AP}1$ in subsystem (7) to be able to obtain a compatible behaviour with system (1).

**Fig. 11:**
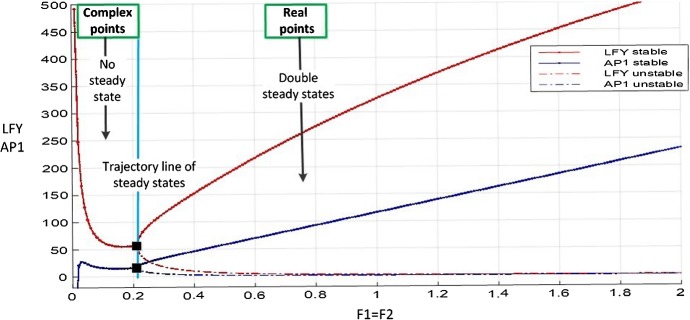
$\textit{LFY}$ and $\textit{AP}1$ with values of $F_i$ from 0 to 2 and ($F_1=F_2$) in Eq. (9). The saddle–node occurring at $F_1=0.21156$ divides the existence of steady states into two regions. Two steady states occur right of the trajectory line for each value of *F* where one of them is a stable state, shown with solid line, and others are unstable, shown with dashed line. There are no positive steady states on the left of the trajectory line. The black points on the trajectory line show the degenerated stable steady-state values of $\textit{LFY}$ and $\textit{AP}1$ (Color figure online)

**Subsystem 3.** For subsystem 3, we distinguish two cases. In the first case, $\textit{FT}$-$\textit{FD}$ and $\textit{FT}$ are assumed to equally inhibit/activate $\textit{LFY}$ and $\textit{AP}1$ by using the same maximum transcription rate. A saddle–node bifurcation occurs at the value $F_1=F_2 = 0.21156$; hence, two distinct positive steady states exist for all larger values, as illustrated in Fig. 11. The numerical solutions confirm that the actions of $\textit{FT}$ on $\textit{AP}1$ and $\textit{FT}$-$\textit{FD}$ on $\textit{LFY}$ do not start any interaction for the flowering of Arabidopsis until the inhibition value of $F_1 = 0.21156$. This situation is represented in the left-hand side of the trajectory line in Fig. 11 where there is no steady state. (The solutions of (14) for ${\bar{x}}_2$ are complex.) The right-hand side of the trajectory line on this figure shows the stable and unstable steady states, indicating that the Arabidopsis flowering is in process.

**Fig. 12:**
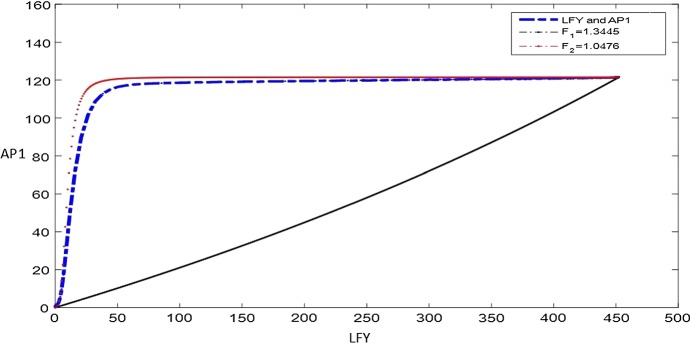
Nullclines in (9) with $F_1=1.3445$ and $F_2 = 1.0476$ with their intersection corresponding to the $AP1-LFY$ steady state. The comparison with the numerical solution of system (1) is also given as the blue dotted line (Color figure online)

**Fig. 13:**
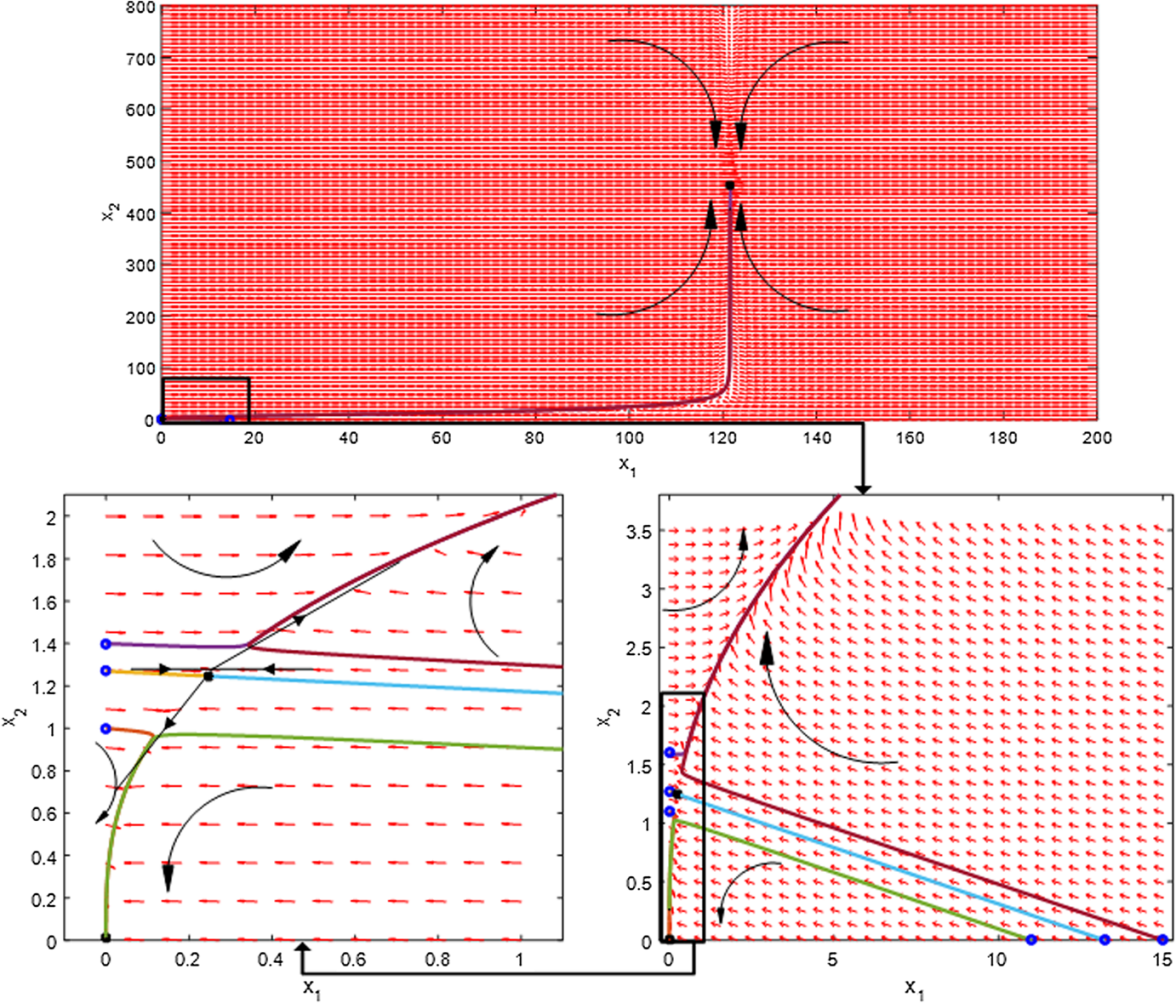
Phase plane of the $\textit{AP}1$ and $\textit{LFY}$ for the obtained values $F_1=1.3445$ and $F_2=1.0476$ in system (7). Black dots represent the steady states, and blue dots on the *x*-axis and *y*-axis represent the initial conditions $(AP1,LFY=0)$ and $(AP1=0,LFY)$ for the coloured lines, respectively. Light blue and dark yellow lines show the eigenvectors at the saddle node. Below and above these two lines, the trivial and non-trivial stable steady states are reached, respectively. The curves with red and black colours show trajectories of the system, where their arrows represent the direction of the phase flow (Color figure online)

In the second case, we assume $F_1$ and $F_2$ may be different from each other. In this circumstance, the best match with system (1) is for $F_1=1.3445$ and $F_2=1.0476$. These results are obtained from LHS and RHS of (8) by using the estimated parameters from Table 2 and matching the steady-state values of $x_1$ and $x_2$ from Table 3. Comparison with the solution of system (1) is given in Fig. 12, showing that subsystem 3 captures well the behaviour of the full model (1) after $\textit{FT}$-$\textit{FD}$ and $\textit{FT}$ start activating $\textit{LFY}$ and $\textit{AP}1$, respectively. In this case, by considering the direction of the flow $\left( \dfrac{d x_{1}}{dt}, \dfrac{dx_{2}}{dt}\right)$ in the $(x_1, x_2)$ phase plane of system (7) for the obtained value $F_1$ and $F_2$ (Fig. 13), it can be explicitly seen that the trivial and non-trivial upper steady states are stable, while the lower non-trivial one is unstable.

The unstable steady state can be regarded as the threshold values of the concentrations for the flowering of *Arabidopsis thaliana*. As a consequence, if flowering is processing for some time which means the concentrations have already reached their threshold values for the flowering, then the values of concentrations can move away from an unstable steady state and converge to a non-trivial stable one, which shows the same flowering behaviour as in the full model.

On the other hand, the initial value of the concentrations over threshold influences the flowering time of the *Arabidopsis thaliana* which can be seen in detail in the following figure.

**Fig. 14:**
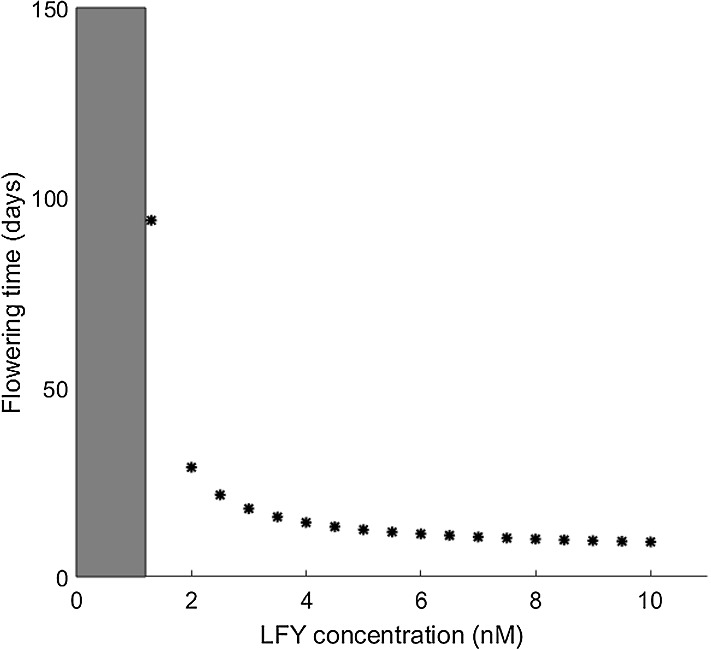
Influence of initial $\textit{LFY}$ concentration on the flowering time of *Arabidopsis thaliana*, predicted using the two-dimensional motif (7) (with $F_1 = 1.3445$ and $F_2 = 1.0476$). The initial value of $\textit{AP}1$ is chosen just over the threshold (0.24 nM). There is no flowering seen for initial $\textit{LFY}$ concentrations below 1.25 nM. The flowering time can thus be accelerated depending on the chosen initial value of $\textit{LFY}$ above this threshold

As can be seen in Fig. 14, in which the $$\textit{AP}1$$ value is chosen just over its threshold (0.24*nM*), if the initial value of $$\textit{LFY}$$ is lower than 1.25*nM*, then no flowering is observed. This is in agreement with the findings of van Dijk and Molenaar (2017), according to which mutants with knocked-down $$\textit{LFY}$$ may not flower. A lower threshold of $$\textit{LFY}$$$$\approx 1nM$$ was estimated for the flowering of these mutants (see van Dijk and Molenaar 2017, SI Figure 9).

## Stochastic Models of Motifs

To obtain more realistic representations of the behaviour of biological systems, it is appropriate to work with stochastic differential equations (SDEs), which can be obtained by incorporating noise terms into deterministic models. The aim of this section is to introduce and study for the first time SDEs for the behaviour of Arabidopsis flowering.

There are several ways for obtaining a SDEs model. Manninen et al. (2015, 2006) introduced a few different approaches in their papers for incorporating stochasticity into the deterministic models. For example, stochasticity can be incorporated into reaction rates, rate constants or into concentrations by using the chemical Langevin equation. In this study, we integrate stochasticity into reaction rates, starting from the following general form of stochastic nonlinear differential equations,

16 $$\begin{aligned} dX(t) = F(t, X(t)) dt + G(X(t)) dW(t), \end{aligned}$$

and consider additive (stochasticity into rate of each variable) and multiplicative (stochasticity into each reaction rate) white noise forms for *G*(*X*(*t*)), following Mackey and Nechaeva (1994). Both types are described and analysed in the subsections below.

### Stochastic Motifs with Additive White Noise

A general Itô formulation of a system of stochastic differential equations with additive white noise form can be written as

17 $$\begin{aligned} dX(t) = F(t, X(t)) dt + G dW(t), \end{aligned}$$

where the stochastic component *GdW* is added into the rate of each variable. Here, $G=diag[\sigma _1,\cdots ,\sigma _m]$ describes a nonnegative real constant diagonal matrix with parameters $\sigma _j$, $\{j=1,\cdots ,m\}$ and *W*(*t*) is represents an *m*-dimensional standard Brownian motion or Wiener process over $t \in [0,T]$. The general solution of equations (17) can be written as:

$$\begin{aligned} X(t) = X(0) + \int _0^t F(s,X(s))ds + \sum _{j=1}^{m}\int _0^t G_{j} dW_j(s). \end{aligned}$$

The stochastic version of motif (7) can then be written as:

18 $$\begin{aligned} dx_1(t)= & {} \left[ f_1(x_2(t)) - d_1x_1(t) \right] dt + \sigma _1 dW_1(t), \nonumber \\ dx_2(t)= & {} \left[ f_2(x_1(t)) - d_2x_2(t) \right] dt + \sigma _2 dW_2(t), \end{aligned}$$

where $\sigma _{1},\sigma _{2}$ are real constants, and $W_{1}$ and $W_{2}$ are independent standard Wiener processes with increments $dW_i(t) = W_i(t+\varDelta _t)-W_i(t)$, $i=1,2$, and each independent random variable satisfies $dW_i\sim \sqrt{\varDelta t}{\mathcal {N}}(0,1)$. Hill functions $f_1$ and $f_2$ are defined as

$$\begin{aligned} f_1(x_2)=\dfrac{\beta _{1}F_{2}x_{2}^{3}}{x_2^3+K_{1}^{3}}, \quad f_2(x_1)=\dfrac{\beta _{4}F_{1}x_{1}}{x_1+K_{4}}, \end{aligned}$$

and the parameters are the same as in previous sections. For numerical implementations with additive white noise, the Euler–Maruyama method with fixed time step $\Delta t$ is used to solve this Ito SDEs model,

19 $$\begin{aligned} x_i(t+\Delta t) = x_i(t) + F_i(t, x(t)) \Delta t + \sigma _i dW_i . \end{aligned}$$

The deterministic model (7) has three steady states: two of them are stable with a trivial and a non-trivial solution, and one is an unstable, trapped between these two stable steady states. The behaviour of this system depends on the initial conditions of the concentrations. If their initial values are lower than the unstable steady state (sub-threshold value of system (7) for flowering of Arabidopsis), then system will certainly reach the trivial solution which means values are insufficient for triggering process of Arabidopsis flowering. Therefore, flowering of the Arabidopsis will not occur. If their initial values are larger than the unstable steady state, the flowering of this seed will proceed, being attracted by the non-trivial stable steady states of the concentrations.

On the other hand, the behaviour of the stochastic model (18) is more complex and depends on the initial conditions and the amount of noise in each of the concentrations. So, it is not certain whether it reaches non-trivial (passing the sub-threshold for the flowering) or trivial (non-flowering) stable equilibria, a phenomenon known as ”stochastic switching” (Ullah and Wolkenhauer 2011). We show the behaviour of stochastic model (18) with a time-varying histogram to see the change of the behaviour. The initial values are fixed as (0.2, 1.2), which lie between unstable and trivial stable steady states for the parameter values from Table 2. The implementation has been performed 100 times with a fixed constant noise of $5\%$ ($\sigma _i=0.05$).

**Fig. 15:**
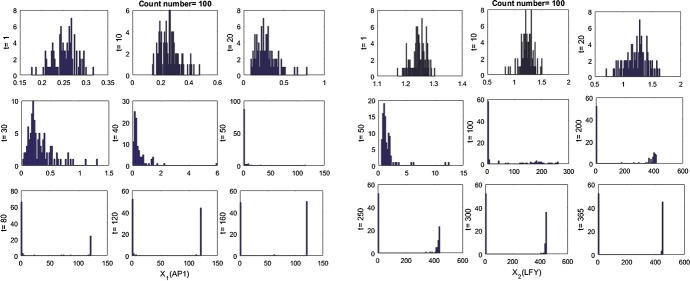
Temporal histogram progress for 100 simulations of the stochastic model (18) for $\textit{AP}1$ (left) and $\textit{LFY}$ (right) using an initial condition of $(x_1, x_2)=(0.2, 1.2)$, just below the unstable steady state and a $5\%$ constant white noise

As can be seen from Fig. 15, stochasticity can change the behaviour of the system. The solutions are initially concentrated around the initial values, and then, they are separated into two different realisations. At the end, they converged around either trivial or non-trivial stable solutions with a considerable proportion. This shows that successful solutions for the Arabidopsis flowering can be obtained by using stochastic equations system even if the initial values are under the threshold value.

**Fig. 16:**
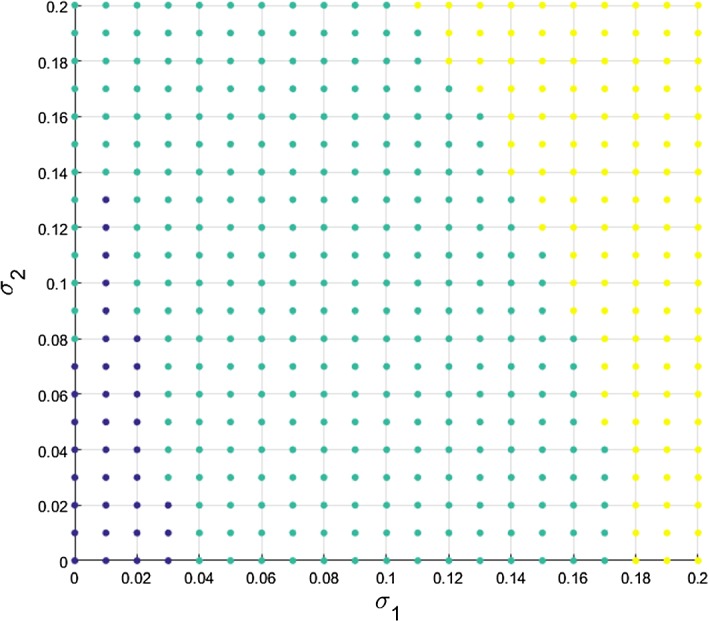
Proportion of successful solutions of the SDEs of Arabidopsis flowering model (18) with random initial condition within 5% of the unstable solution, depending on the noise parameters $\sigma _i$. Blue, green and yellow dots represent success rations of the flowering process for less than $50\%$, between 50 and $70\%$, and more than $70\%$, respectively (Color figure online)

We also consider the effect of the different $\sigma$ values on the stochastic system (18). If we look at the initial values of the concentrations ($x_{init}$) around the unstable steady state within $5\%$ range, $0.95{\bar{x}}<x_{init}<1.05{\bar{x}}$, we obtain the results presented in Fig. 16.

### Stochastic Motifs with Multiplicative White Noise

In contrast to the previous subsection, where the possibility of successful flowering was depending only on the amount of noise terms and initial values of the concentrations, here we assume that the amplitude of noise depends on the state of the system. More precisely, stochastic perturbations of the variables around their equilibrium values are assumed to be of white noise type and proportional to the distances of $AP1 (x_1)$ and $LFY (x_2)$ from the steady-state values ${\bar{x}}_1$ and ${\bar{x}}_2$. The question whether the dynamical behaviour of model (7) is influenced by stochastic effects is investigated by looking at the asymptotic stochastic stability of equilibrium points.

This leads to the following stochastic differential system of the Arabidopsis flowering

20 $$\begin{aligned} dx_1(t)= & {} \left[ f_1(x_2(t)) - d_1x_1(t) \right] dt + \sigma _1 (x_1(t)-{\bar{x}}_1) dW_1(t), \nonumber \\ dx_2(t)= & {} \left[ f_2(x_1(t)) - d_2x_2(t) \right] dt + \sigma _2 (x_2(t)-{\bar{x}}_2) dW_2(t), \end{aligned}$$

where again $\sigma _{i}$ are positive constants, and $W_{i}$ are independent standard Wiener process components, $dW_i\sim \varDelta (t){\mathcal {N}}(0,1)$, with increments $\varDelta W_i(t) = W_i(t+\varDelta _t)-W_i(t)$, $i=1,2$.

The aim is to determine the flowering domain of the stochastic motifs with multiplicative white noise. These can be obtained by using a Lyapunov function approach, centred at the origin or at a non-trivial steady state of the system. This allows to obtain necessary stability conditions which depend on the noise parameters $\sigma _i$.

Let us show that the trivial solution $x=0$ of system (20) is locally asymptotically stable in probability. Using a stochastic stability approach from Khasminskii (2011), we derive that there exists a noise-dependent domain around ${\bar{x}}=0$ for which asymptotic stability holds. This domain thus corresponds to non-flowering conditions for the *Arabidopsis thaliana* GRN modelled by system (20).

#### Theorem 3

The equilibrium point ${\bar{x}}=0$ of system (20) is locally asymptotically stable in probability if the conditions

- $$0 \le \sigma _i < \sqrt{2d_i}$$, $$i=1,2$$,

are satisfied.

#### Proof

3. Let ${\bar{x}}=0 \in D \subset {\mathbb {R}}^2$ be an equilibrium point of the stochastic differential equations system (20) where *D* is defined as a positive neighbourhood of this point. Let us define a positive definite function *V*

21 $$\begin{aligned} V(x)= & {} \frac{1}{2}(\theta x_{1}^{2}+x_{2}^{2}), \end{aligned}$$

where $\theta$ is a strictly positive constant. Applying a differential operator *L* to *V*(*x*), which is acting on the function *V* as

22 $$\begin{aligned} LV = \frac{\partial V}{\partial t} + \sum _{i=1}^n f_i\frac{\partial V}{\partial x^i}+ \frac{1}{2} \sum _{i,j=1}^n G_{ij} \frac{\partial ^{2}V}{\partial x^i x^j}, \quad G_{ij} = \sum _{i_1}^k g_{i m} g_{j m}, \end{aligned}$$

gives the following expression for system (20),

23 $$\begin{aligned} LV(x)= & {} \theta x_1(f_1(x_2) -d_1x_1) + x_2(f_2(x_1)-d_2x_2) + \frac{1}{2}(\theta \sigma _1^{2}(x_{1}-{\bar{x}}_1)^{2}\nonumber \\&+\, \sigma _2^{2}(x_{2}-{\bar{x}}_2)^{2}) \nonumber \\= & {} \theta x_1(f_1(x_2) -d_1x_1) + x_2(f_2(x_1)-d_2x_2) + \frac{1}{2}(\theta \sigma _1^{2}x_{1}^{2}+ \sigma _2^{2}x_{2}^{2}), \qquad \end{aligned}$$

where ${\bar{x}}_1={\bar{x}}_2=0$. We consider the leading terms in some positive neighbourhood around $(x_1, x_2) = (0,0)$ of (23) up to the second-order Taylor expansion, which are

24 $$\begin{aligned} LV(x) \approx -\dfrac{\theta (2d_1-\sigma _1^{2})}{2}x_1^2 - \dfrac{(2d_2-\sigma _2^{2})}{2}x_2^2 + \dfrac{\beta _4F_1}{K_4}x_1x_2. \end{aligned}$$

Using Young’s inequality in (24), $\pm x_1x_2 \le \dfrac{1}{2}(\varepsilon x_1^2+\dfrac{1}{\varepsilon }x_2^2), \forall \epsilon >0$, we obtain,

25 $$\begin{aligned} LV(x) \le -\dfrac{\theta (2d_1-\sigma _1^{2})}{2}x_1^2 - \dfrac{(2d_2-\sigma _2^{2})}{2}x_2^2 + \dfrac{\beta _4F_1}{K_4}\dfrac{1}{2}\left( \varepsilon x_1^2+\dfrac{1}{\varepsilon }x_2^2\right) . \end{aligned}$$

By grouping $x_1^2$ and $x_2^2$, we find

26 $$\begin{aligned} LV(x)\le & {} -x_1^2 \left[ \dfrac{\theta (2d_1-\sigma _1^2)}{2}-\dfrac{\beta _4F_1\epsilon }{2K_4} \right] - x_2^2 \left[ \dfrac{(2d_2-\sigma _2^2)}{2}-\dfrac{\beta _4F_1}{2K_4\epsilon } \right] . \end{aligned}$$

The system is locally and asymptotically stable in probability if $LV(x)<0$; therefore, the following inequalities are required,

27 $$\begin{aligned} \left( 2d_1 - \sigma _1^2 \right) \theta - \left( \dfrac{\beta _4F_1}{K_4} \right) \epsilon> & {} 0, \end{aligned}$$

28 $$\begin{aligned} (2d_2-\sigma _2^2)\epsilon - \left( \dfrac{\beta _4F_1}{K_4} \right)> & {} 0. \end{aligned}$$

In particular, this implies that

29 $$\begin{aligned} \sigma _1^2<2d_1, \quad \sigma _2^2<2d_2. \end{aligned}$$

Then, combining inequalities (27) and (28), we find,

30 $$\begin{aligned} \dfrac{\beta _4 F_1}{\left( 2d_2 - \sigma _2^{2} \right) K_4}< \epsilon < \dfrac{\theta K_4 \left( 2d_{1} - \sigma _1^{2} \right) }{\beta _4F_1}. \end{aligned}$$

Since all parameters are positive, this inequality can be rearranged as

31 $$\begin{aligned} \theta> \left( \dfrac{\beta _4F_1}{K_4}\right) ^2\dfrac{1}{\left( 2d_1-\sigma _1^{2}\right) \left( 2d_2-\sigma _2^{2}\right) } > 0, \end{aligned}$$

which shows that for any $\sigma _1, \sigma _2$ satisfying $\sigma _i < \sqrt{2 d_i}$ one can choose a suitable positive value of $\theta$ such that *V* is a local Lyapunov function of the system. Thus, the origin is locally asymptotically stable in probability. $\square$

## Conclusion

In this paper, we considered a dynamic model of Arabidopsis flowering introduced by Valentim et al. (2015). This model is reconstructed with Hill functions to emphasise the importance of these functions and their effects on the concentrations. An analytical study of the deterministic model and its steady state for the full system was performed. The stability analysis was used to establish the conditions for initiating the transition to flowering. The steady states are calculated numerically with the estimated parameters taken from Valentim et al. (2015). The analysis results have shown that the system has only one positive stable steady state and that the time for which $\textit{AP}1$ reaches the steady state is in agreement with the observed flowering time between 20 and 30 days. The Routh–Hurwitz criterion has been used to provide local stability conditions which characterise the existence of this stable steady state; details are given in “Appendix”.

Given the complexity of the system, more precise conditions have been formulated by considering subsystems which focus on the dynamics of essential elements. According to our analysis for the full system, three genes, $\textit{SOC}1$, $\textit{LFY}$ and $\textit{AP}1$, have a strong effect on the flowering of Arabidopsis. The network has been simplified by decoupling. Analytical solution of the simplified system is still difficult; however, it illustrates specific pathways of inhibition and activation. By using these pathways, we reconstruct three different subsystems suggested in Jaeger et al. (2013) and Pullen et al. (2013). This allowed us to derive necessary and sufficient conditions for the existence of the positive steady states of these subsystems that represent the dynamics and cooperativity of the Arabidopsis flowering time regulation system. The most important floral identity genes, $\textit{AP}1$ and $\textit{LFY}$, are used to investigate the flowering where they are regulating each other, and the results are confirmed by experiments (Liljegren et al. 1999). The necessary and sufficient conditions for the local stability of the deterministic model have then been determined analytically, and the stability ranges are established with the estimated parameters and compared with the numerical solutions. The numerical results have confirmed that these subsystems can capture the essential behaviour of the full model by estimating the $\textit{FT}$-$\textit{FD}$ inhibition/activation effects on $\textit{LFY}$ and $\textit{AP}1$, and also they help to investigate the conditions (threshold values) for the initiation of flowering, which cannot be obtained from the full model. Moreover, stochastic motifs, which are extended from the deterministic ones by adding additive and multiplicative white noise terms, have been developed to obtain more realistic description of gene effects and their interactions on the behaviour of Arabidopsis flowering. The effects of stochasticity on the steady-state regimes have been observed. The numerical solutions show that the flowering behaviour of the system does not only depend on the initial values, state variables and parameters of the stochastic system but also the amount of noise terms, where the noise can change behaviour of the stability region from non-flowering to flowering through a stability switch even if the initial values are lower than the threshold values.

Our analyses, being in a good agreement with the experimental findings, bring further insights into the roles of $\textit{LFY}$ and $\textit{AP}1$ and provide the opportunity to explore different pathways for flowering.

## Figures and Tables

**Fig. 17:**
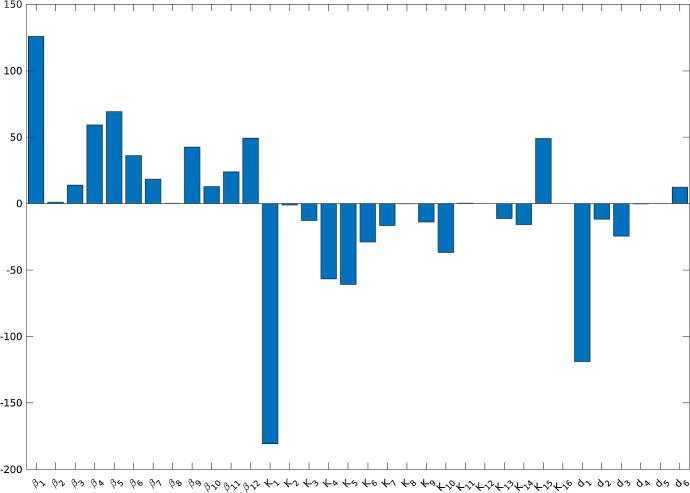
Maximum value of the normalised parameter sensitivity index of system (1) with respect to $\textit{AP}1$

**Fig. 18:**
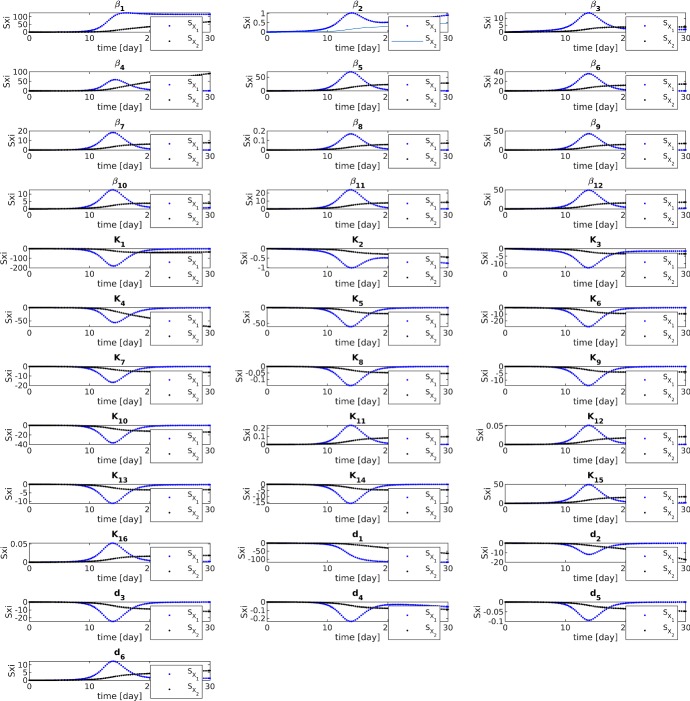
Parameter sensitivity analysis of system (1) highlighting the influence of each parameter onto $\textit{AP}1$ and $\textit{LFY}$

## A Analytical Results

### A.1 Steady State of the Full Model

- From (3a), by substituting ${\bar{x}}_4$ and ${\bar{x}}_6$ from (3d) and (3f), respectively, we find
  32 $$\begin{aligned} {\bar{x}}_1 = \dfrac{p_1{\bar{x}}_2^{4}+p_2{\bar{x}}_2^{3}+p_3{\bar{x}}_2+p_7U_1}{p_4{\bar{x}}_2^{4}+p_5{\bar{x}}_2^{3}+p_6{\bar{x}}_2+p_7}, \end{aligned}$$
  where the constants are defined by,
  33 $$\begin{aligned}&p_1 = (d_4K_{2}+\beta _{10})(\beta _{1}+d_1U_1)+\beta _{2}\beta _{10}, \ \ p_2 = d_4K_{2}K_{13}(\beta _{1}+d_1U_1), \nonumber \\&p_3 = K_{1}^{3}(\beta _{2}\beta _{10}{+}d_1U_1(d_4K_{2}{+}\beta _{10})), \ \ p_4 = {d_1}(d_4K_{2}+\beta _{10}), \ \ p_5 = {d_1}d_4K_{2}K_{13}, \nonumber \\&p_6 = {d_1}K_{1}^{3}(d_4K_{2}+\beta _{10}), \ \ p_7 = {d_1}d_4K_{1}^{3}K_{2}K_{13} \ \hbox { and}\nonumber \\&U_1 = \dfrac{\beta _{3}u}{d_1(K_{3}+u)}. \end{aligned}$$
  Equation (32) establishes the link between ${\bar{x}}_1$ and ${\bar{x}}_2$.
- From (3b), by substituting ${\bar{x}}_5$ from (3e) and ${\bar{x}}_1$ from (32), we find
  34 $$\begin{aligned}&\dfrac{s_{10}{\bar{x}}_2^5+(s_{11}-s_6){\bar{x}}_2^4-s_7{\bar{x}}_2^3+s_{12}{\bar{x}}_2^2+(s_{13}-s_8){\bar{x}}_2-s_9}{(s_{10}{\bar{x}}_2^4+s_{11}{\bar{x}}_2^3+s_{12}{\bar{x}}_2+s_{13})}\nonumber \\&\quad = \dfrac{s_1{\bar{x}}_3^2+s_2{\bar{x}}_3}{(s_3{\bar{x}}_3^2+s_4{\bar{x}}_3+s_5)}, \end{aligned}$$
  where the constants $s_i$ are given by
  $$\begin{aligned}&s_1 = \beta _{6}\beta _{11}+d_5\beta _{5}K_{6}+\beta _{5}\beta _{11}, s_2 = \beta _{6}\beta _{11}K_{5}+d_5\beta _{5}K_{6}K_{14},\\&s_3 = d_2(d_5K_{6}+\beta _{11}), \\&s_4 = d_2(d_5K_{5}K_{6}+\beta _{11}K_{5}+d_5K_{6}K_{14}), \ s_5 = d_2d_5K_{5}K_{6}K_{14}, \ s_6 = \beta _{4}p_1, \\&s_7 = \beta _{4}p_2, \ s_8 = \beta _{4}p_3, \ s_9 = \beta _{4}U_1p_7, \ s_{10} = d_2(K_{4}p_4+p_1),\\&s_{11} = d_2(K_{4}p_5+p_2), \\&s_{12} = d_2(K_{4}p_6+p_3) \hbox { and } s_{13} = d_2(K_{4}p_7+U_1p_7). \end{aligned}$$
- From (3c), by substituting ${\bar{x}}_4$, ${\bar{x}}_5$ and ${\bar{x}}_6$ from (3d), (3e) and (3f), respectively, we obtain
  35 $$\begin{aligned} \dfrac{n_{1}{\bar{x}}_3^{3}+n_{2}{\bar{x}}_3^{2}+n_{3}{\bar{x}}_3}{n_{1}{\bar{x}}_3^{2}+n_{4}{\bar{x}}_3+n_{5}}=\dfrac{m_{6}{\bar{x}}_2}{m_{7}+m_{8}{\bar{x}}_2}, \end{aligned}$$
  where the constants are given by
  $$\begin{aligned}&m_1 = \beta _{8}\beta _{11}\kappa _{11}(x_7)\kappa _{12}(x_{8}), \ \ m_2 = d_3d_{5}K_{8}+d_{3}\beta _{11}, \ \ m_{3} = d_{3}d_{5}K_{8}K_{14}, \\&m_{4} = \beta _{7}\kappa _{11}(x_{7})\kappa _{12}(x_{8}), \ \ m_{5} = d_{3}K_{7}, \ \ m_{6} = u\beta _{9}\beta _{10}\kappa _{11}(x_{7})\kappa _{12}(x_{8}), \\&m_{7} = d_{3}d_{4}K_{9}K_{13}(K_{10}+u), \ \ m_{8} = d_{3}(d_{4}K_{9}+\beta _{10})(K_{10}+u), \ \ n_{1} = m_{2}d_{3}, \\&n_{2} = m_{3}d_{3}+m_{2}m_{5}-m_{1}d_{3}-m_{2}m_{4}, \ \ n_{3} = m_{3}m_{5}-m_{1}m_{5}-m_{3}m_{4}, \\&n_{4} = m_{3}d_{3}+m_{2}m_{5}, \ \ n_{5} = m_{3}m_{5}. \end{aligned}$$

### A.2 Linear Stability Analysis of the Full Model

Linearisation of the nonlinear system (1) is required to analyse the local stability of this dynamic model at its steady state points $({\bar{x}}_1,{\bar{x}}_2,{\bar{x}}_3,{\bar{x}}_4,{\bar{x}}_5,{\bar{x}}_6)$. To linearise the system with time delay, the following equation is introduced

36 $$\begin{aligned} \dot{X}=J_{0}X+J_{\tau }X(\tau ) \end{aligned}$$

to describe the behaviour in a neighbourhood of the steady-state point, where $X=(x_{1},x_{2},x_{3},x_{4},x_{5},x_{6})$, $X(\tau )=(x_{1}(\tau ),x_{2}(\tau ),x_{3}(\tau ),x_{4}(\tau ),x_{5}(\tau ),x_{6}(\tau ))$. In this equation, $J_{0}$ and $J_{\tau }$ are Jacobians of the system with respect to non-delayed and delayed variables, respectively,

$$\begin{aligned} J_{0}=\left( \dfrac{\partial f_{i}}{\partial x_{j}} \right) \Bigg |_{X=X(\tau )={\bar{x}}} \ \ \hbox { and }\ \ J_{\tau }=\left( \dfrac{\partial f_{i}}{\partial x_{j}(\tau )} \right) \Bigg |_{X=X(\tau )={\bar{x}}}. \end{aligned}$$

The matrix form of the linearised system (36) is given as

37 $$\begin{aligned} \begin{bmatrix} \dot{x_{1}}\\ \dot{x_{2}}\\ \dot{x_{3}}\\ \dot{x_{4}}\\ \dot{x_{5}}\\ \dot{x_{6}} \end{bmatrix}=\underset{\mathbf {J_{0}}}{\underbrace{ \begin{bmatrix} -d_{1}&A&0&B&0&0\\ C&-d_{2}&D&0&E&0\\ 0&0&F-d_{3}&G&H&0\\ 0&K&0&-d_{4}&0&0\\ 0&0&L&0&-d_{5}&0\\ 0&0&0&0&0&-d_{6} \end{bmatrix} } } \begin{bmatrix} x_{1}\\ x_{2}\\ x_{3}\\ x_{4}\\ x_{5}\\ x_{6} \end{bmatrix} + \underset{\mathbf {J_{{\varvec{\tau }}}}}{\underbrace{ \begin{bmatrix} 0&0&0&0&0&M\\ 0&0&0&0&0&0\\ 0&0&0&0&0&N\\ 0&0&0&0&0&0\\ 0&0&0&0&0&0\\ 0&0&0&0&0&0 \end{bmatrix} } } \begin{bmatrix} x_{1}(\tau )\\ x_{2}(\tau )\\ x_{3}(\tau )\\ x_{4}(\tau )\\ x_{5}(\tau )\\ x_{6}(\tau ) \end{bmatrix},\nonumber \\ \end{aligned}$$

where the following notation is used,

$$\begin{aligned}&A=\beta _{1}{\gamma _{1}^3}'({\bar{x}}_{2}), \ \ B=\beta _{2}{\gamma _{2}}'({\bar{x}}_{4}), \ \ C=\beta _{4}{\gamma _{4}}'({\bar{x}}_{1}), \ \ D=\beta _{5}{\gamma _{5}}'({\bar{x}}_{3}), \\&E=\beta _{6}{\gamma _{6}}'({\bar{x}}_{5}), \\&F=\beta _{7}{\gamma _{7}}'({\bar{x}}_{3}){\kappa _{11}}(x_{7}){\kappa _{12}}(x_{8}), \ \ G=\beta _{9}{\gamma _{9}}'({\bar{x}}_{4}){\gamma _{10}}({\bar{x}}_{6}){\kappa _{11}}(x_{7}){\kappa _{12}}(x_{8}), \\&H=\beta _{8}{\gamma _{8}}'({\bar{x}}_{5}){\kappa _{11}}(x_{7}){\kappa _{12}}(x_{8}), \ \ K=\beta _{10}{\gamma _{13}}'({\bar{x}}_{2}), \ \ L=\beta _{11}{\gamma _{14}}'({\bar{x}}_{3}), \\&M=\beta _{3}{\gamma _{3}}'({\bar{x}}_{6}), \\&\hbox { and }\quad N={\gamma _{10}}'({\bar{x}}_{6}){\gamma _{9}}({\bar{x}}_{4}){\kappa _{11}}(x_{7}){\kappa _{12}}(x_{8}). \end{aligned}$$

Here, ${\gamma _j}'({\bar{x}}_i)$, for $j=1,\ldots ,16$ and $i=1,\ldots ,6$ denote derivatives of ${\gamma _j}$ with respect to ${x_i}$ at the steady-state point. The determinant below is introduced to obtain the characteristic equation,

38 $$\begin{aligned} det(J_{0}+e^{-\lambda \tau }J_{\tau }-\lambda I)= \begin{vmatrix} -d_{1}-\lambda&A&0&B&0&Me^{-\lambda \tau }\\ C&-d_{2}-\lambda&D&0&E&0\\ 0&0&F-d_{3}-\lambda&G&H&Ne^{-\lambda \tau }\\ 0&K&0&-d_{4}-\lambda&0&0\\ 0&0&L&0&-d_{5}-\lambda&0\\ 0&0&0&0&0&-d_{6}-\lambda \end{vmatrix} =0,\nonumber \\ \end{aligned}$$

where *I* is an identity matrix. This gives the following characteristic equation,

39 $$\begin{aligned} P_1(\lambda )= & {} (d_{6}+\lambda ) P_2(\lambda ), \end{aligned}$$

where

40 $$\begin{aligned} P_2(\lambda )= & {} \Big [(d_{3}+\lambda -F)(d_{5}+\lambda )-HL\Big ]\nonumber \\&\Big [(d_{1}+\lambda )(d_{2}+\lambda )(d_{4}+\lambda )-(d_{4}+\lambda )AC-BCK\Big ]\nonumber \\&-\Big [(d_{1}+\lambda )GK((d_{5}+\lambda )D+EL)\Big ]=0. \ \end{aligned}$$

It is clear that $\lambda =-d_6<0$ is a root of this characteristic equation. Thus, we now only focus on the stability of $P_2(\lambda )$ by using the Routh–Hurwitz stability criterion (Gantmacher et al. 1960).

#### Theorem 4

A steady state of the nonlinear system (1) is locally asymptotically stable iff all the roots of the polynomial

41 $$\begin{aligned} P_2(\lambda )= & {} \lambda ^5+a_1\lambda ^4+a_2\lambda ^3+a_3\lambda ^2+a_4\lambda +a_5, \end{aligned}$$

have negative real parts, that is iff the following conditions are satisfied,

$$\begin{aligned}&a_i>0, \ \ i=1, \ldots , 5, \\&a_1a_2a_3 + a_1a_5 \> \ a_3^2+a_1^2a_4, \ \hbox { and }\\&(a_1a_4-a_5)(a_1a_2a_3+a_1a_5-a_3^2-a_1^2a_4) \ > \ a_5(a_1a_2-a_3)^2, \end{aligned}$$

where

$$\begin{aligned} a_1= & {} d_1+d_2+d_3+d_4+d_5-F ,\\ a_2= & {} -HL - d_5F + d_3d_5 + (d_3+d_5-F)(d_1+d_2+d_4)\\&- AC + d_1d_2 + d_1d_4 + d_2d_4 , \\ a_3= & {} - (d_1+d_2+d_4)(HL+d_5F-d_3d_5) \\&- (d_3+d_5-F)(AC-d_1d_2-d_1d_4-d_2d_4)\\&- (d_4AC + BCK + DGK - d_1d_2d_4) ,\\ a_4= & {} (HL+d_5F-d_3d_5)(AC-d_1d_2-d_1d_4-d_2d_4) \\&- (d_3+d_5-F)(d_4AC+BCK-d_1d_2d_4) - (EGKL+(d_1+d_5)DGK) ,\\ a_5= & {} (d_4AC+BCK-d_1d_2d_4)(HL+d_5F-d_3d_5) - (d_1d_5DGK+d_1EGKL) . \end{aligned}$$

Otherwise, the steady state of the system is unstable.

For the stability condition, all roots of $P_2(\lambda )$ must have negative real part. By using the Routh–Hurwitz scheme (Saeed 2008) for the 5-*th* degree characteristic polynomial $P_2(\lambda )$,

All roots will have negative real part iff the coefficients $a_i$, ($i=1,..,5$), $b_1$, $b_2$, $c_1$, $c_2$, $d_1$ and $e_1$, are all bigger than zero. Assuming $a_i>0$, ($i=1,..,5$), the stability conditions of $P_2(\lambda )$ can be derived as

$$\begin{aligned}&a_1a_2>a_3, \ \ \ a_1a_4>a_5, \ \ \ a_1a_2a_3+a_1a_5>a_3^2+a_1^2a_4, \quad \hbox { and }\\&(a_1a_4-a_5)(a_1a_2a_3+a_1a_5-a_3^2-a_1^2a_4)>a_5(a_1a_2-a_3)^2. \end{aligned}$$

These conditions show the validity of Theorem 4. For the parameters given in Table 2 and the initial input values in Table 4, the steady state (3) of the nonlinear system (1) satisfies all conditions of Theorem 4;

where

$$\begin{aligned}&a_1=0.9764, \ a_2=0.1026, \ a_3=0.0021, \ a_4=1.2003e^{-5}, \ a_5=9.9659e^{-9}, \\&a_1a_2-a_3=0.098, \ a_1a_4-a_5=1.171e^{-5}, \\&a_1a_2a_3+a_1a_5-a_3^2-a_1^2a_4=1.9817e^{-4} \quad \ \hbox { and }\\&(a_1a_4-a_5)(a_1a_2a_3+a_1a_5-a_3^2-a_1^2a_4)-a_5(a_1a_2-a_3)^2=2.2247e^{-9}. \end{aligned}$$

### A.3 Linear Stability Analysis of the Simplified Model

The matrix form of the linearised simplified system is given as

42 $$\begin{aligned} \begin{bmatrix} \dot{x_{1}}\\ \dot{x_{2}}\\ \dot{x_{3}} \end{bmatrix}=\underset{{\mathbf {J}}}{\underbrace{ \begin{bmatrix} -d_{1}&{\mathcal {A}}&0 \\ {\mathcal {B}}&-d_{2}&{\mathcal {C}} \\ 0&{\mathcal {D}}&{\mathcal {E}}-d_{3} \end{bmatrix} } } \begin{bmatrix} x_{1}\\ x_{2}\\ x_{3} \end{bmatrix}, \end{aligned}$$

where the following notation is used,

$$\begin{aligned}&{\mathcal {A}}=\dfrac{V_2S_3}{(S_2{\bar{x}}_2+S_3)^2}+\dfrac{3V_1S_1^3{\bar{x}}_2^2}{({\bar{x}}_2^3+S_1^3)^2}, \ \ {\mathcal {B}}=\dfrac{V_3S_4}{({\bar{x}}_1+S_4)^2}, \\&\quad {\mathcal {C}}=\dfrac{V_4S_5}{({\bar{x}}_3+S_5)^2}+\dfrac{V_5S_7}{(S_6{\bar{x}}_3+S_7)^2}, \\&\quad {\mathcal {D}}=\dfrac{V_6S_9}{(S_8{\bar{x}}_2+S_9)^2}, \ \ {\mathcal {E}}=\dfrac{V_7S_{10}}{({\bar{x}}_3+S_{10})^2}+\dfrac{V_8S_{12}}{(S_{11}{\bar{x}}_3+S_{12})^2}. \ \ \end{aligned}$$

The determinant $det(J-\lambda I)=0$ gives the following characteristic equation.

43 $$\begin{aligned} P(\lambda )= & {} (d_1+\lambda ) \Big [ (d_2+\lambda )({\mathcal {E}}-(d3+\lambda )) + {\mathcal {C}}{\mathcal {D}} \Big ] - {\mathcal {A}}{\mathcal {B}}({\mathcal {E}}-(d3+\lambda )) \nonumber \\= & {} \lambda ^3 + \lambda ^2(d_1+d_2+d_3-{\mathcal {E}}) + \lambda \Big [ d_1d_2+d_1d_3+d_2d_3-(d_1+d_2){\mathcal {E}}-{\mathcal {A}}{\mathcal {B}}-{\mathcal {C}}{\mathcal {D}} \Big ] \nonumber \\&+ d_1d_2d_3 + {\mathcal {A}}{\mathcal {B}}{\mathcal {E}} - d_1d_2{\mathcal {E}} - d_1{\mathcal {C}}{\mathcal {D}} - d_3{\mathcal {A}}{\mathcal {B}} = 0. \end{aligned}$$

By using the Routh–Hurwitz scheme for the 3-*th* degree characteristic polynomial $P(\lambda )$,

we obtain the stability conditions of $P(\lambda )$ as follows:

$$\begin{aligned} a_i>0, i=1,..,3 \hbox { and } a_1a_2>a_3. \end{aligned}$$

All roots will have negative real part iff the coefficients of $P(\lambda )$ and $b_1$ are all bigger than zero.

These conditions show the validity of Theorem 2. For the parameters given in Table 2, and the initial input values in Table 4, steady state of the nonlinear simplified system (5) satisfies all conditions of Theorem 2 where

$$\begin{aligned} a_1=0.9679, \quad \ a_2=0.0943, \quad \ a_3=0.0013 \quad \ \hbox { and } \quad b_1=a_1a_2-a_3=0.093. \end{aligned}$$

### A.4 Stability Analysis of Deterministic Motifs

The characteristic equation of Jacobian matrix (12) is obtained as,

44 $$\begin{aligned} P(\lambda )={\lambda }^2+(d_{1}+d_{2}) {\lambda } + d_{1}d_{2}-\dfrac{nd_1d_2K_{1}^nK_{4}}{({\bar{x}}_1+K_{4})({\bar{x}}_2^n+K_{1}^n)}, \end{aligned}$$

where $F_1$ and $F_2$ have been eliminated using Eq. (8). For asymptotic stability, we require that $Re\lambda <0$. Therefore, the necessary and sufficient conditions for local stability are $\mathrm {tr}(J)<0$ and $\mathrm {det}(J)>0$. The first condition is already satisfied, while the second one gives

45 $$\begin{aligned} \mathrm {det}(J)=d_1d_2-\dfrac{nd_1d_2K_{1}^nK_{4}}{({\bar{x}}_1+K_{4})({\bar{x}}_2^n+K_{1}^n)}>0. \end{aligned}$$

Substituting ${\bar{x}}_1$ from Eq. (8) into inequality (45), we find

46 $$\begin{aligned} {\bar{x}}_2^n>\dfrac{(n-1)d_1K_{1}^nK_{4}}{(d_1K_{4}+\beta _1F_2)}. \end{aligned}$$

Eliminating ${\bar{x}}_2^n$ from (11), we obtain

47 $$\begin{aligned} {\bar{x}}_2^{n}=\dfrac{\omega _3F_1F_2{\bar{x}}_2^{n-1}-\omega _4}{(\omega _1+\omega _2F_2)}=\dfrac{\beta _1\beta _4F_1F_2{\bar{x}}_2^{n-1}-d_1d_2K_{1}^nK_{4}}{d_2(d_1K_4+\beta _1F_2)}, \end{aligned}$$

and applying equation (47) to inequality (46),

48 $$\begin{aligned} \dfrac{\beta _1\beta _4F_1F_2{\bar{x}}_2^{n-1}-d_1d_2K_{1}^nK_{4}}{d_2(d_1K_4+\beta _1F_2)}>\dfrac{(n-1)d_1K_{1}^nK_{4}}{(d_1K_{4}+\beta _1F_2)}, \end{aligned}$$

gives the necessary and sufficient condition for stability as given in inequality (13).

## B Tables

See Table 4.

**Table 4:** Independent input variables with the initial values (Valentim et al. 2015)

Input variables	Described in the model	Initial input values (nM)
SVP-meristem	$SVP_m \rightarrow x_7$	3.507
FLC-meristem	$FLC_m \rightarrow x_{8}$	0.423
SVP-leaves	$SVP_l \rightarrow x_9$	156.947
FLC-leaves	$FLC_l \rightarrow x_{10}$	1.047

## C Parameter Sensitivity

Parameter sensitivity analysis is an approach to determine the influence of each parameters on a mathematical model output. This analysis helps to figure out the most significant parameters, which have the most important effect on the behaviour of a model.

For a given dynamical system

$$\begin{aligned} \dot{x} = f(x,p), \quad x(t_0) = x_0, \end{aligned}$$

the local parameter sensitivity matrix *S* is obtained through the solution of an augmented system composed of the system and the sensitivity equations

$$\begin{aligned} \dot{S} = \frac{\partial f}{\partial x} S + \frac{\partial f}{\partial p}, \quad S(t_0) = 0. \end{aligned}$$

This gives an assessment of the time-dependent influence of each parameter on the solution *x*. Focusing on determining which parameters most contribute to the AP1 dynamics around the flowering period, we obtain the sensitivity graphs in Figs. 17 and 18. They show that the most dominant parameters, namely $\beta _1, \beta _4, \beta _5, K_1, K_4, K_5$ and $d_1$, belong to the first two equations.
